# Physiologically relevant 3D CRISPR screening enhances mechanistic insight into chemical toxicity compared to 2D screening

**DOI:** 10.1016/j.tox.2026.154422

**Published:** 2026-02-03

**Authors:** Chanhee Kim, Zhaohan Zhu, Abderrahmane Tagmount, W. Brad Barbazuk, Rhonda Bacher, Christopher D. Vulpe

**Affiliations:** a Center for Human and Environmental Toxicology, Department of Physiological Sciences, College of Veterinary Medicine, University of Florida, Gainesville, FL, United States; b Department of Biostatistics, University of Florida, FL, United States; c Department of Biology, University of Florida, FL, United States; d University of Florida Genetics Institute, University of Florida, FL, United States

**Keywords:** **(1–7):** functional toxicogenomics, 3D CRISPR screening, NAMs, Spheroid, Genetic variability, Chemical toxicity, Doxorubicin, Anthracyclines

## Abstract

CRISPR-based approaches can complement other genomics-based toxicology studies by enabling causal interrogation of gene function modulating chemical-induced toxicity. Moreover, CRISPR screens enable scalable and systematic identification of functional pathways involved in cellular response to chemical exposure. Cell-based functional toxicogenomics approaches using CRISPR provide a potential powerful tool for the development of mechanism-driven new approach methodologies (NAMs) for toxicodynamic and toxicokinetic hazard screening to enable more effective risk assessment. To improve the physiological relevance of in vitro functional toxicogenomics, we developed a three-dimensional (3D) CRISPR screening platform using HepG2/C3A spheroids cultured in a continuously rotating bioreactor (ClinoStar). We evaluated the potential utility of a 3D CRISPR screen as compared to conventional 2D screen using a custom CRISPR sgRNA library representing common loss-of-function genetic variants in the human population and exposure to the well characterized DNA damaging toxicant, doxorubicin. The 3D platform identified more genes and pathways in which variants have previously been associated with doxorubicin toxicity in clinical studies than the 2D system. These results support the utility of 3D CRISPR screening to identify physiologically relevant genetic determinants underlying chemical toxicity.

## 1. Introduction

Systems biology approaches, such as multiple omics platforms, are widely applied in toxicological research to aid in the mechanistic understanding of chemical toxicity including identification of gene products and biological pathways underlying toxicological endpoints (Alexander-Dann et al., 2018; Harrill et al., 2021; Beale et al., 2022). However, such approaches can rely on correlative data and often lack evidence of functional relationships or causal links between observed omics changes and phenotypes(Hasin et al., 2017). Since its discovery, CRISPR(Jinek et al., 2012) genome engineering tools are being widely adopted to interrogate gene function across biomedical research and complement other genomic approaches(Barrangou and Doudna, 2016; Asmamaw and Zawdie, 2021). CRISPR-based functional genomics screening (CRISPR screening) has transformed our ability to systematically interrogate gene function(Arroyo et al., 2016; Chang et al., 2020; Rossiter et al., 2021; Awah et al., 2022). By enabling genome-wide or targeted genetic perturbations with high efficiency, CRISPR screening provides a powerful and versatile platform to identify genetic determinants of diverse cellular processes at scale. In toxicology, CRISPR screens offer an unbiased approach to uncover the molecular mechanisms underlying chemical-induced toxicity and relevant adverse outcomes(Gáytan and Vulpe, 2014; Sobh, Loguinov, Stornetta, et al., 2019; Sobh, Loguinov, Yazici, et al., 2019; Wang et al., 2022). Despite its potential utility, large-scale CRISPR screening applications in toxicology remain limited(Sobh and Vulpe, 2019; Sobh, Loguinov, Stornetta, et al., 2019; Sobh et al., 2021). In addition, most CRISPR-based cell based toxicology studies, including our own work, rely on 2D cell culture systems(Doench, 2018; Bock et al., 2022), which may limit the physiological relevance to human toxicity assessment(Duval et al., 2017; Kapałczyńska et al., 2018).

3D cell models can offer significant advantages over conventional 2D cultures in toxicological research, as they can better recapitulate human physiological conditions and toxicant responses, including improved xenobiotic metabolism that is more similar to *in vivo* metabolism (Gunness et al., 2013; Juarez-Moreno et al., 2022; Tang, Shi, et al., 2023). In addition, 3D cultures are hypothesized to more closely mimic the *in vivo* microenvironment than 2D cultures through enhanced cell-cell and cell-extracellular matrix (ECM) interactions, tissue-like architecture, and gene and protein expression patterns that resemble *in vivo* states(Gunness et al., 2013; Kapałczyńska et al., 2018; Tang, Shi, et al., 2023). These *in vivo*-comparable characteristics has led to the adoption of various 3D models to produce more physiologically relevant cellular responses than their 2D counterparts even when the same cell lines are used(Kim et al., 2024, Anon, n.d.). 3D models may improve the identification of whole organism relevant responses critical for effective toxicological risk assessment. However, identifying functional gene–toxicant interactions in physiologically relevant human models remains challenging due to the lack of appropriate experimental systems. The recent shift toward reducing animal testing under the framework of new approach methodologies (NAMs) further underscores the importance of 3D *in vitro* models in toxicology(Stresser et al., 2024; Beilmann et al., 2025). While several applications of 3D CRISPR screening have been demonstrated in cancer research(Han et al., 2020; Takahashi et al., 2020), such approaches have not yet been established or critically evaluated for toxicological studies.

Here, we developed a 3D CRISPR screening system using HepG2/C3A human liver spheroids for toxicological applications to enhance the physiological relevance of the conventional 2D functional toxicogenomics approach. First, we performed time-course 3D CRISPR screens to identify functional genetic components influencing spheroid growth compared to 2D culture in normal growth media. Next, we applied this approach to a chemical toxicity study using doxorubicin (Doxo) as a model chemical to compare the performance of 3D and 2D screens in identifying genes and pathways associated with Doxo-induced toxicity. Doxo was chosen due to its well-characterized DNA damage–inducing mechanism of action(Thorn et al., 2011; Nicoletto and Ofner, 2022), providing a reference framework for mechanistic comparison. Doxo is primarily associated with cardiotoxicity(Chen et al., 2023; Fonoudi et al., 2024; Robert Li et al., 2024), but also has documented hepatotoxic effects (Prasanna et al., 2020; Lai et al., 2022; Yeerkenbieke et al., 2025), which are less well characterized. We used custom CRISPR sgRNA libraries (PopVarLoF) representing common human loss-of-function (LoF) genes (mean allele frequency > 0.1 % in all individuals catalogued in the genome aggregation database (gnomAD V3.0) (Karczewski et al., 2020)). The PopVar LoF sgRNA library enables systematic testing of a subset of genes with common LoF alleles in the human population which could contribute to a better understanding of population variability in human response to toxicant. Using this approach, we identified both known and previously unrecognized genes harboring common LoF genetic variants potentially modulating doxorubicin exposure-related phenotypes in people. Overall, we demonstrated that a 3D CRISPR screening system can provide a physiologically relevant and scalable platform for functional toxicogenomics, enabling the discovery of candidate causal genetic determinants and pathways underlying genetic variability in toxicity in people.

## 2. Materials and methods

### 2.1. HepG2/C3A cell culture in 2D monolayer and 3D spheroid

The HepG2/C3A cells were directly purchased from the American Type Culture Collection (ATCC, Manassas, Virginia). Cells were maintained and passaged following ATCC’s recommended protocol. Minimum Essential Medium (MEM, Gibco) was supplemented with 10 % fetal bovine serum (FBS, Thermo Fisher Scientific, Waltham, MA), 1X MEM Non-Essential Amino Acids Solution 100X (Gibco, added for HepG2/C3A) and 1X Antibiotic-Antimycotic 100X (Gibco). Cells were cultured in a humidified incubator (air has water vapor to protect the cells and medium, Forma^™^ Series II Water-Jacketed CO2 Incubator, Thermo Scientific^™^) with 5 % CO_2_ at 37 °C. For 3D spheroid cultures, we followed the protocol from our previous study(Kim et al., 2024) except that dissociation and reassembly of spheroids were added to the 3D CRISPR screening procedure which is described in 2.3. ClinoReactors and ClinoStar (CelVivo) were used to generate and maintain HepG2/C3A spheroids under a continuously rotating bioreactor system for up to 30 days.

### 2.2. Cytotoxicity of doxorubicin in 2D monolayer and 3D spheroid

The cytotoxicity of 2D HepG2/C3A cells exposed to Doxo was assessed using a range of nominal concentrations (0.4–100 μM) for 72 h by measuring ATP levels using the CellTiter-Glo2.0 cell viability assay kit (Promega, Madison, WI) following the manufacturer’s instruction. For 3D spheroid cytotoxicity assays, 10 spheroids on Day 7 were assessed using a range of nominal concentrations (0.4–100 μM) for 72 h by measuring ATP levels using the CellTiter-Glo 3D cell viability assay kit (Promega, Madison, WI) following the manufacturer’s instructions. The luminescence signals were read on a Synergy H1 microplate reader (BioTek Instruments, Winooski, VT). Relative luminescence signals were calculated and converted to % of viable cells compared to controls. The % values were used to determine the 25 % inhibitory concentrations (IC_25_) for Doxo in 2D and 3D (72 h) using GraphPad Prism (version 10.1.0) with the dose-response sigmoid function.

### 2.3. CRISPR screens

Lentiviral packaging of the two custom PopVarLoF sgRNA plasmid libraries (SET1 and SET2)(Kim et al., 2025) was carried out as previously described(Sobh, Loguinov, Stornetta, et al., 2019; Sobh, Loguinov, Yazici, et al., 2019) (a list of sgRNA sequences is in Table S1). The resulting PopVar LoF lentiviral libraries were functionally tittered in HepG2/C3A cells to determine the amount of virus required to obtain a multiplicity of infection of 0.3 for each library. For large-scale transduction of these libraries for 2D and 3D CRISPR screens, HepG2/C3A cells were seeded in four 12-well culture plates (1 ×10^6^ cells/well) prior to lentiviral transduction. After 24 h, polybrene (Sigma) was added to each well to a final concentration of 8 μg/mL, along with 0.5 μl of each PopVar LoF lentiviral library (SET1 and SET2), followed by centrifugation at 33 °C at 1000xG for 2 h (transduction). After incubation at 37°C for 30 h, the media containing the lentiviral transduction mixture was replaced with 1 mL of the growth media in each well. After 24 h of recovery, we selected transduced cells with puromycin (2 μg/mL) until non-transduced controls were eliminated, ensuring that only successfully transduced cells remained. After 3 days of recovery and expansion from puromycin selection, the cells were used either for 2D monolayer screens or 3D spheroid screens. To generate transduced 3D spheroids, we cultured the transduced HepG2/C3A cells using the 3D culture method described in 2.1. Notably, the number of transduced cells corresponding to approximately 500-fold (500X) the total PopVar LoF library size was maintained throughout both 2D and 3D CRISPR screens (Doench, 2018). Two types of CRISPR screens were conducted in this study, as described below:

#### 2.3.1. Time-course screens 3D vs. 2D

Both transduced initial 2D monolayer cells and 3D spheroids (2D_Day 0 and 3D_ Day 0) were cultured in a CO_2_ incubator (T75 flasks) and ClinoStar (ClinoReactors) continuously for up to 30 days. Time-course analysis samples were harvested on Day 20 and Day 30 (2D_Day 20, 2D_Day 30, 3D_ Day 20, and 3D_ Day 30). Culture media were changed every 3–4 days for both 2D and 3D systems.

#### 2.3.2. Chemical toxicity screens (doxorubicin) 3D vs. 2D

Our previous study(Kim et al., 2024) indicated an appropriate time window for exposure in 3D CRISPR screens (Day 3 to Day 10) of the HepG2/CA3 cells grown as spheroids in the ClinoStar system. HepG2/C3A 3D spheroids exposed to Doxo IC_25_ (0.4 μM, 72 h) were dissociated into single cells using Accutase (STEMCELL Technologies) on Day 6 (when exposure was suspended), reassembled into spheroids on Day 7, and then re-exposed to Doxo at same dose until Day 14. This adjustment to the 3D exposure regimen ensured comparability with the 2D Doxo exposure condition in terms of the number of cell doublings under chemical exposure (selection). For 2D exposure, we performed a pre-screen time-course treatment with the initial benchmark IC_25_ (0.18 μM, 72 h) dose over 14 days by passaging the treated cells every 3–4 days to refine the exposure conditions for the screen and to assess longer-term cellular responses. Doxo-exposed samples were harvested on Day 14 (7 cell doublings) for both 2D and 3D, with corresponding no-exposure controls.

### 2.4. Next Generation Sequencing (NGS) preparation and sequencing

Genomic DNA was extracted from 1.6 × 10^6^ cells of each sample using the Quick-DNA Midiprep Plus Kit (ZYMO Research-D4075) following the manufacturer’s protocols. Amplicons for NGS Illumina sequencing were generated using pairs of universal CRISPR-FOR1 forward primer and CRISPR-REV**#** reverse primers (**#**: 1–48) each specific to a corresponding sample (Table S2). Amplicons for each sgRNA in each sample were then pooled and gel purified using the QIAquick Gel Extraction Kit (Qiagen) and quantified using the Qubit HS dsDNA assay (Thermo Scientific). Equimolar amounts of each amplicon library were multiplexed into a single pool, following the procedure described in previous studies(Sobh, Loguinov, Stornetta, et al., 2019; Sobh, Loguinov, Yazici, et al., 2019). The Illumina sequencing was carried out at the Interdisciplinary Center for Biotechnology Research (ICBR), University of Florida at Gainesville, using the NovaSeqX paired 150 bp high-throughput platform (Illumina).

### 2.5. Data analysis and bioinformatics

All FASTQ files were first assessed for sequencing quality using FastQC (http://www.bioinformatics.babraham.ac.uk/projects/fastqc). Read counts were generated from FASTQ files using the MaGeCK count command(Li et al., 2014), which aligned reads to the provided PopVar LoF sgRNA library file and produced a sgRNA-level count matrix for downstream analysis. Differential selection analysis was performed using DESeq2 (version 1.40.2)(Love et al., 2014) in R (version 4.3.3) to identify differentially selected sgRNAs between the baseline (Day 0) and time-course points (Day 14 and Day 28). For each comparison of time-course screens (2D_Day 20 vs. Day 0, 2D_Day 30 vs. Day 0, 3D_ Day 20 vs. Day 0, and 3D_ Day 30 vs. Day 0), three Day 0 samples and two time-course samples from the respective time points were analyzed. For each comparison of chemical toxicity screens (2D_Doxo vs. 2D_Cont, 3D_Doxo vs. 3D_Cont), four 2D samples and two 3D samples were analyzed. The DESeq2 library size factor for normalization was calculated using the estimateSizeFactors function with the safe-harbor sgRNAs set as the control genes (Table S1). Safe-harbor control sgRNAs have been demonstrated to be a more appropriate baseline for normalization(Morgens et al., 2017; Chen et al., 2018) and thus safe-harbor sgRNAs were used for size-factor estimation and normalization in DESeq2 across the biological and technical replicates for each dose/time point. To further incorporate effect-size information in a data-driven manner, we derived empirical Log_2_FC cutoffs for selection of candidate genes. Specifically, safe-harbor sgRNAs were used to empirically approximate the null (background) distribution of Log_2_FC and defined lower (-Log_2_FC) and upper (+Log_2_FC) effect-size cutoffs as the 2.5^%^ and 97. 5 % quantiles of the Log_2_FC distribution, respectively. These empirical cutoffs for Log_2_FC were used to identify significantly depleted or enriched sgRNA in each screen, rather than using a fixed Log_2_FC cutoff across experiments. For time-course screens, decreased sgRNA abundance indicated a growth-disadvantage phenotype, whereas increased abundance indicated a growth-advantage phenotype. For chemical toxicity screens, decreased sgRNA abundance indicated a sensitivity to Doxo, whereas increased abundance indicated a resistance to Doxo. Since the PopVarLoF library is composed of two separate libraries (SET1 and SET2) and each library has one sgRNA per gene (i.e., two sgRNAs per gene), a gene was deemed significantly depleted or enriched at a dose/time point if at least one of its two sgRNAs was significantly differentially selected.

### 2.6. Functional enrichment analysis

Gene Ontology-Biological Process (GO-BP) and Kyoto Encyclopedia of Genes and Genomes (KEGG) pathway enrichment analyses were performed using the Log_2_FC values of candidate genes, either sensitive or resistant to Doxo exposure in 2D and 3D screens. Enrichment analyses were conducted using the clusterProfiler tool implemented in SRPLOT software(Tang, Chen, et al., 2023) (SRplot, accessed May 2025). STRING database(Szklarczyk et al., 2023) (STRING: functional protein association networks, accessed Mar 2025) was used to cluster the gene products of candidate genes sensitive or resistant to Doxo exposure in 2D and 3D screens using a default setting (accessed on Mar 2025). We annotated each cluster containing two or more gene products based on GO-BP terms and KEGG pathways available in the STRING database.

### 2.7. ClinPGx doxorubicin phenotype data

The ClinPGx database (ClinPGx, accessed Sep. 2025) was queried to identify pharmacogenomic associations for anthracycline drugs, including doxorubicin, daunorubicin, epirubicin, and idarubicin, which share a common DNA damage–inducing mechanism of action. Curated datasets containing reported associations between genetic variants and drug-related phenotypes were downloaded from the database. To generate a non-redundant dataset, overlapping entries across the four anthracycline drugs were identified and removed before downstream comparisons.

## 3. Results

### 3.1. Time-course 3D CRISPR screen identified distinct genes conferring growth disadvantage or advantage from those identified in the 2D screen

**Fig. 1.**
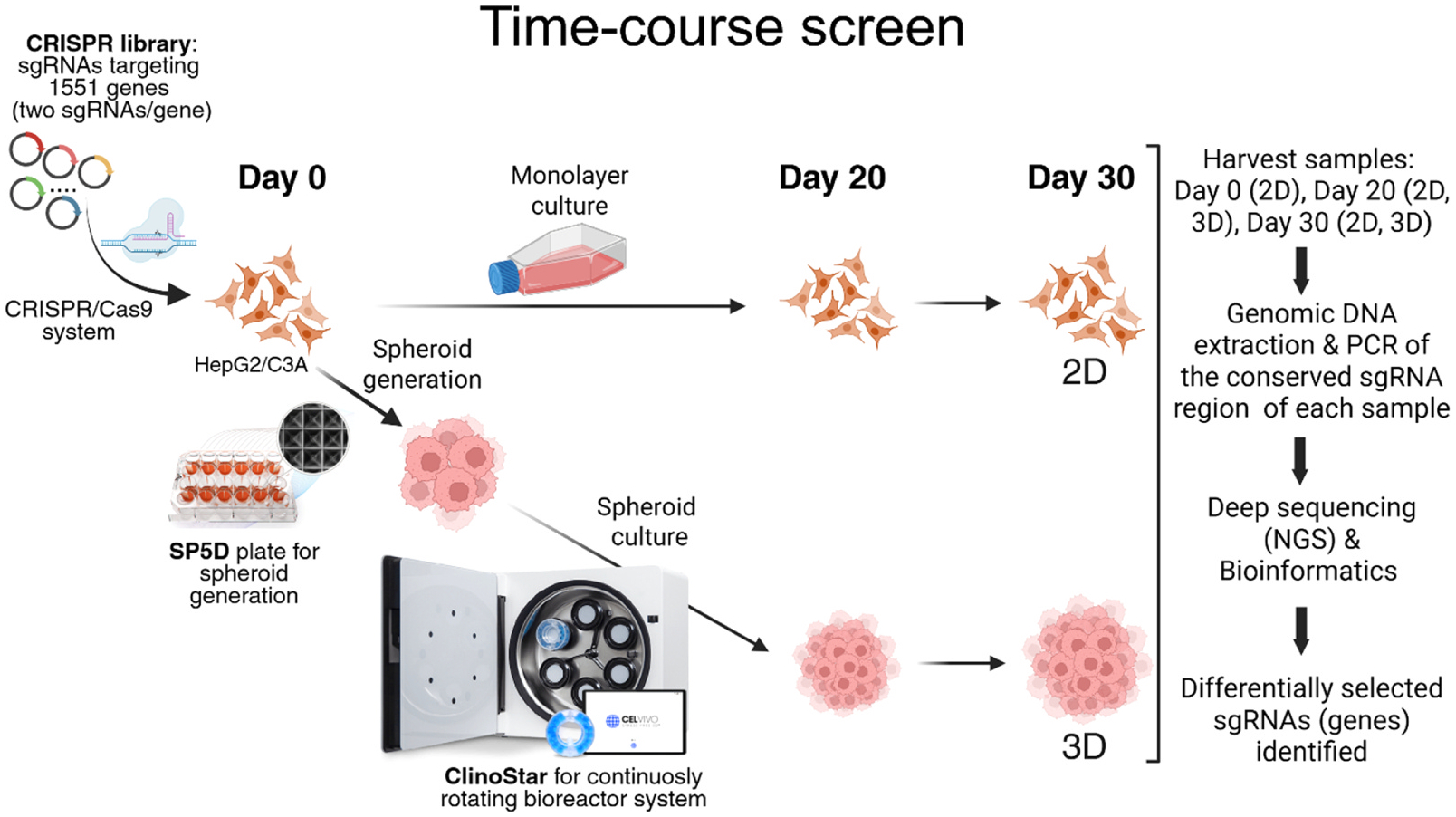
Illustration of the workflow of the time-course 3D CRISPR screening system compared to 2D screening in standard growth conditions. SP5D, Spherical Plate 5D; NGS, next-generation sequencing.

**Fig. 2.**
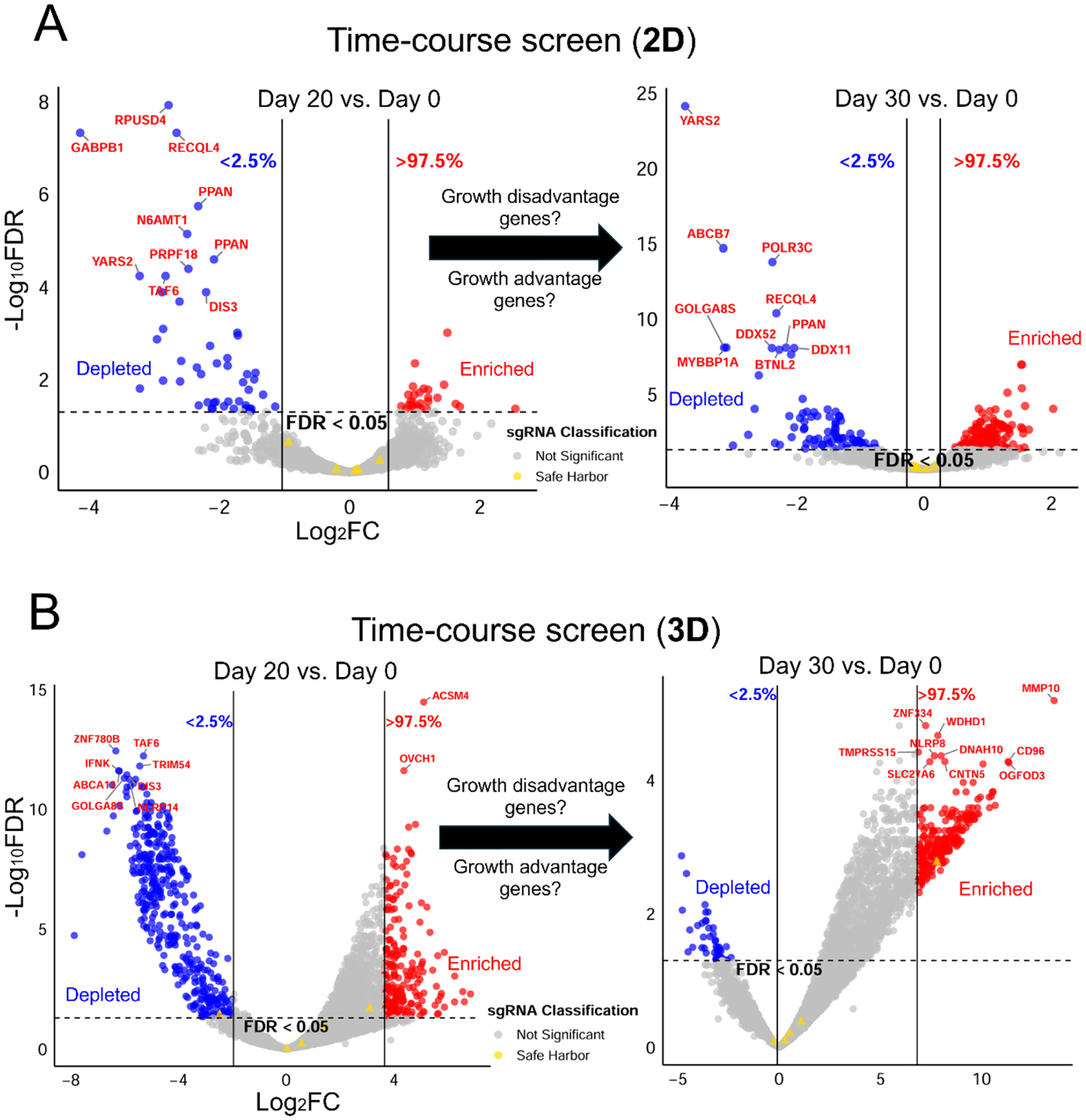
Time-course 3D CRISPR screening system compared to 2D screening in standard growth conditions for each system. Volcano plots show differentially selected candidate genes (depleted in blue; enriched in red) in 2D (A) and 3D (B) time-course CRISPR screens comparing Day 20 vs. Day 0 (left) and Day 30 vs. Day 0 (right). Statistically significant candidate genes (FDR<0.05, and Log_2_FC <2.5 % or >97.5 % of empirical distribution cutoff, see methods for details) are labeled in blue (depleted) and red (enriched). sgRNA classification indicates not significant sgRNAs (grey) and safe harbor sgRNAs (yellow). By comparing two time-course volcano plots, sgRNAs (genes) that, when disrupted, resulted in a consistent growth disadvantage (continuously depleted) or growth advantage (continuously enriched) were identified. In each plot, x-axis indicates log_2_ (fold change) and y-axis indicates −log_10_ (adjusted p-value; FDR).

**Fig. 3.**
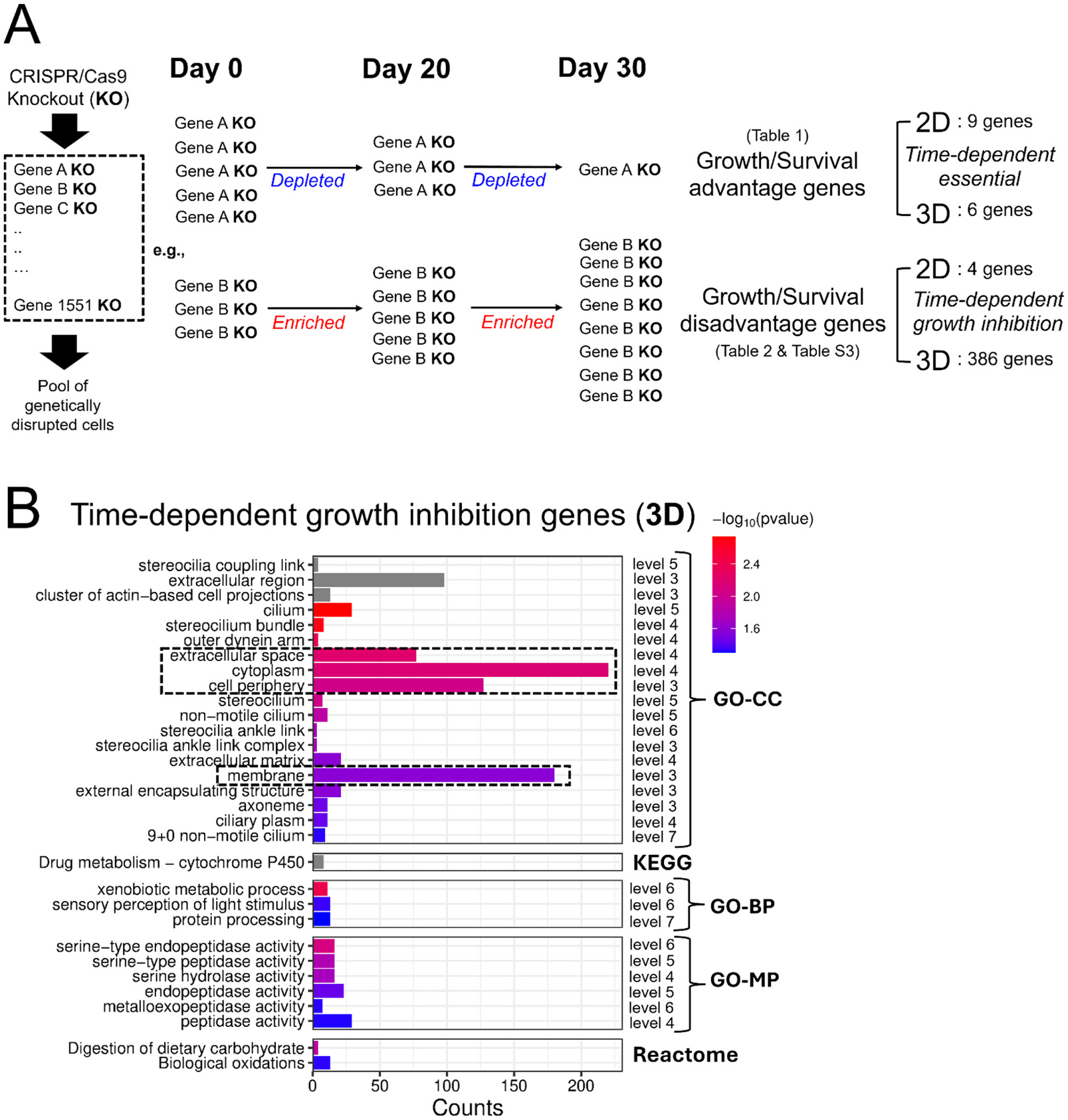
Differentially selected sgRNAs (genes) identified from time-course CRISPR screens of 2D monolayer and 3D spheroid in normal growth media. (A) Illustration of how time-dependent essential (TDE) and time-dependent growth inhibition (TDGI) genes are defined. Both 2D and 3D CRISPR screens were conducted for 30 days. Time-course samples were collected on Day 20 and Day 30 and compared to the Day 0 initial mutant cell population. Gene knockout (KO)s showing statistically significant and consistent depletion over time (based on Log_2_FC values for Day 30 vs. Day 0 and Day 20 vs. Day 0 comparisons) were classified as time-dependent essential (TDE) genes. In contrast, gene KOs showing continuous enrichment were classified as time-dependent growth inhibition (TDGI) genes. (B) Functional enrichment of 3D TDGI genes (386 genes) using STRING protein network analysis. The dashed boxes indicate the terms with more than 50 gene counts and *p-value* less than 0.05. Individual GO terms are annotated with corresponding GO hierarchical levels (level 3–7: specifical terms). GO-CC, Gene Ontology-Cellular Component; GO-BP, Gene Ontology-Biological Process; GO-MP, Gene Ontology-Molecular Function.

To compare the functional requirements for cellular growth and survival of HepG2/C3A in the 3D culture system as compared to in conventional 2D cell culture, we first performed time-course CRISPR screens under normal growth conditions to identify genes that, when disrupted, confer a growth disadvantage or advantage in each system (Fig. 1). We carried out CRISPR screens in HepG2/C3A cultured in 2D and in 3D using a custom CRISPR sgRNA library (in LentiCRISPRv2-Puro (Addgene #52961), an all in one vector containing both the sgRNA and Cas9) representing the ~1551 human genes with common aggregate LoF mutations(mean allele frequency >0.1 % in gnomAD V3.0) in the human population developed in our previous study(Kim et al., 2025). The initial pool of CRISPR mutants (500X representation) of HepG2/C3A after lentiviral transduction and selection was expanded and used to establish either the 3D spheroid cultures or 2D monolayer cultures (Day 0 for both), which were subsequently maintained for 30 days in their respective growth environments (Fig. 1). For 3D culture, we employed the ClinoStar bioreactor system (CelVivo), which supports stable and continuous spheroid growth over extended periods, as demonstrated in our previous study(Kim et al., 2024). Time-course samples (500X representation) were harvested on Day 20 and Day 30 and compared with the Day 0 initial population. The relative abundance of each sgRNA, which is indicative of the abundance of each corresponding targeted mutant cell, was quantified using next-generation sequencing to determine how genetic disruption of each corresponding gene affected cell growth in both 3D and 2D conditions. Decreased sgRNA abundance indicates that disruption of the gene targeted by the sgRNA results in reduced growth or a growth-disadvantage phenotype. In contrast, increased abundance suggests that disruption of the gene targeted by the sgRNA results in enhanced growth, or a growth-advantage phenotype relative to other mutants in the pool. Differentially selected sgRNAs (genes) in 3D and 2D systems were identified across four comparison sets: Day 20 vs. Day 0 (3D), Day 30 vs. Day 0 (3D), Day 20 vs. Day 0 (2D), and Day 30 vs. Day 0 (2D) (Fig. 2A and Fig. 2B). Genes, when disrupted, showing increasing depletion over time, were empirically classified as time dependent essential (TDE) genes, while those showing increasing enrichment were classified as time dependent growth inhibition (TDGI) genes in each respective system (Fig. 2A and Fig. 2B). By comparing relative sgRNA abundance (Log_2_FC values) between time points and the Day 0 baseline, we identified 9 and 6 growth-disadvantage genes in 2D and 3D cultures, respectively, and 4 and 386 growth-advantage genes in 2D and 3D, respectively (Fig. 3A, Tables 1 and 2, Supplementary Table S3). For growth-disadvantage genes, we compared our results with the DepMap gene essentiality database(Tsherniak et al., 2017) (Table 1), which catalogs essential genes (EGs) critical for cell survival across diverse human cell lines in 2D culture. Among the 9 genes identified in 2D, 7 genes (*ABCB7*, *DDX11*, *DDX52*, *DIS3*, *MYBBP1A*, *POLR3C*, and *YARS2*) were previously reported as essential, one gene (*ADAM2*) was non-essential, and one gene (*GOLGA8S*) had no available data in DepMap (Table 1). For 3D time dependent essential genes, only 2 out of 6 genes (*CCDC63* and *TAF6*) were previously identified as essential in 2D cultures, one gene (*FDXACB1*) was non-essential, and 3 genes (*MYH7B*, *NBPF9*, and *TLP1*) had no DepMap data (Table 1). Interestingly, while only 4 genes (*CYP2A13*, *KMT2C*, *OTOP3*, and *ZNF556*) which when targeted resulted in enrichment of the corresponding mutant were detected in the 2D screen, the 3D screen identified a substantially larger set of 355 3D TDGI genes (Table 2). STRING protein network analysis revealed that 3D TDGIs were significantly enriched in the cytoplasm, cell periphery, membrane, and extracellular space (GO-CC terms; >50 genes per term; p < 0.05) (Fig. 3B), which we speculate could include gene products which play an inhibitory role in cellular proliferation in a 3D environment (e.g. cell contact inhibition).

### 3.2. The 3D CRISPR screen identified more candidate genetic modulators of doxorubicin-induced toxicity than the 2D screen

**Fig. 4.**
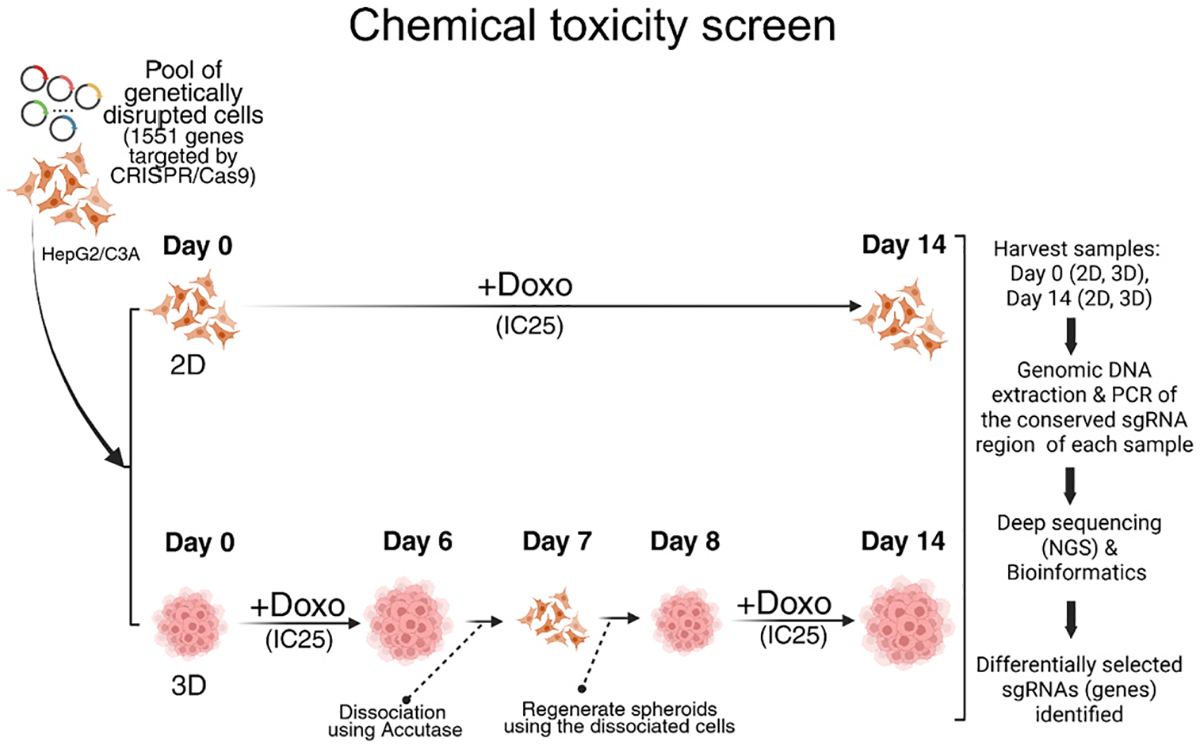
Application of the 3D CRISPR screening system to a chemical toxicity study using doxorubicin (Doxo) as a model chemical. Schematic workflow of the 3D CRISPR screening system for Doxo-induced chemical toxicity screen compared to the 2D system. IC_25_, inhibitory concentration of 25, where a 25 % reduction in cell viability endpoints compared to the control is observed.

**Fig. 5.**
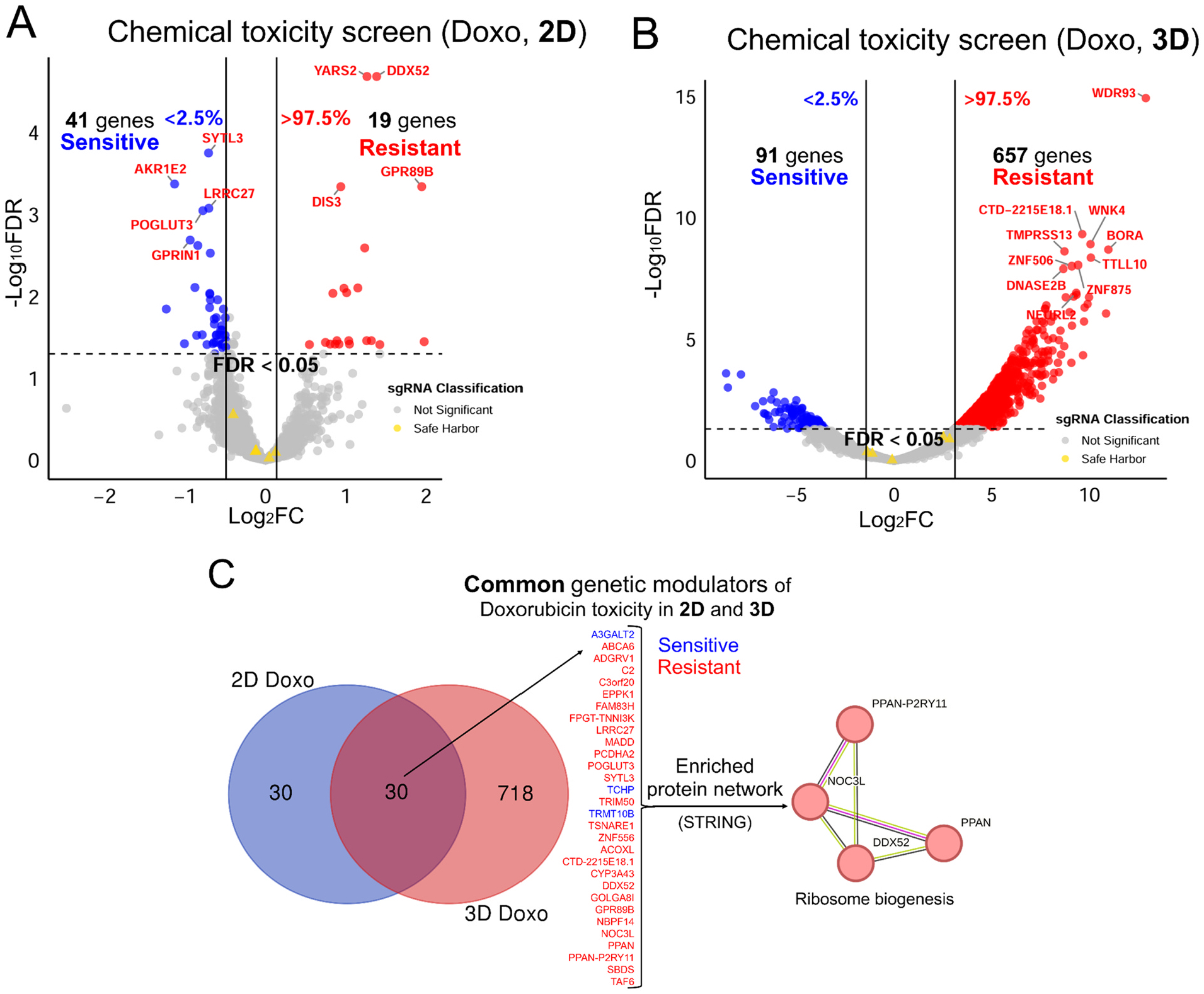
3D CRISPR screening system to a chemical toxicity study using doxorubicin (Doxo) as a model chemical. (A) A volcano plot of 2D Doxo CRISPR screens identifies candidate genes that, when disrupted, result in increased Doxo sensitivity (blue dots) or increased Doxo resistance (red dots) in the CRISPR screens. (B) A volcano plot of 3D Doxo CRISPR screens displays Doxo-sensitive (blue dots) and Doxo-resistant (red dots) candidate genes identified in the screens. (A and B) The top 10 statistically significant candidate genes (FDR<0.05, and Log_2_FC <2.5 % or >97.5 % of empirical distribution cutoff, see methods for details) are labeled and sgRNA classification indicates not significant sgRNAs (grey) and safe harbor sgRNAs (yellow). In each plot, x-axis indicates log_2_ (fold change) and y-axis indicates −log_10_ (adjusted p-value = FDR). (C) Common candidate genes identified in both 2D and 3D screens. A Venn diagram shows the number of common candidate genes. Blue and red colored genes indicate if disruption increases sensitivity or resistance to Doxo-induced cellular toxicity, respectively. An enriched functional protein network of the common candidate genes is visualized via STRING network analysis.

To apply our 3D CRISPR screening system to the study of chemical toxicity mechanisms for comparison to the 2D system, we used doxorubicin (Doxo), which is a well-characterized DNA-damaging agent (Fig. 4). Exposure to the respective IC_25_ (72 h) of Doxo in 3D spheroid and 2D monolayer of HepG2/C3A cells was used to identify candidate genes that, when disrupted, increased sensitivity or resistance to Doxo-induced cellular toxicity (Supplementary Fig. S1). To ensure comparable chemical exposures across 3D and 2D systems the initial mutant cell populations were treated with Doxo for approximately seven cell doublings (14 days, collected on Day 14). For 3D cultures, HepG2/C3A spheroids were treated for 6 days, dissociated, and reassembled into new spheroids to match the effective doubling time of 2D cultures, thereby controlling for previously observed proliferation rate differences(Kim et al., 2024) (Fig. 4, see Methods). Differentially selected sgRNAs (genes) were identified by quantifying relative sgRNA abundance via next-generation sequencing, revealing how genetic perturbations affected Doxo response in 3D versus 2D environments. Decreased sgRNA abundance indicated a sensitivity of the mutant cell with the corresponding gene disruption to Doxo, whereas increased abundance indicated a resistance of the mutant cell with the corresponding gene disruption to Doxo. The 2D Doxo CRISPR screen identified 60 candidate genetic modulators (41 sensitive genes and 19 resistant genes; Fig. 5 A), whereas the 3D Doxo CRISPR screen identified 748 candidate modulators (91 sensitive genes and 657 resistant genes; Fig. 5B). Despite this large difference in the total number of modulators, 30 genes were consistently identified in both 2D and 3D screens, of which 28 genes conferred resistance to Doxo when disrupted (Fig. 5 C). To identify enriched protein networks among the common genetic modulators, we performed STRING protein network analysis, which revealed a single enriched term, Ribosome Biogenesis, comprising four genes (*PPAN-P2RY11*, *NOC3L*, *DDX52*, and *PPAN*) shared between the 2D and 3D screens (Fig. 5 C).

### 3.3. The 3D CRISPR screen of doxorubicin more accurately captured mechanisms relevant to chemical-specific toxicity compared to the 2D screen

**Fig. 6.**
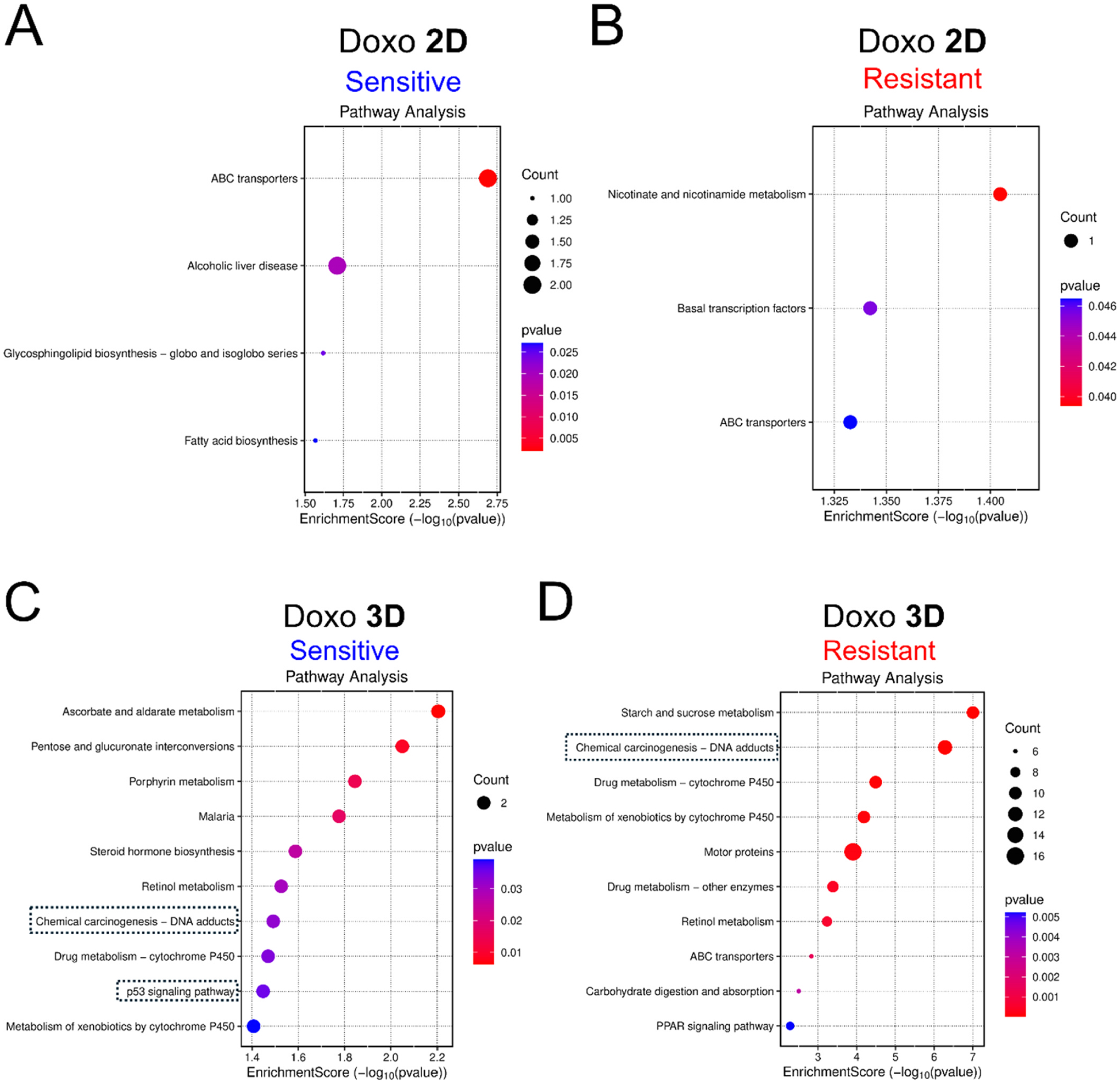
KEGG pathway enrichment of candidate genes modulating doxorubicin (Doxo)-induced cellular toxicity identified in 2D (A and B) and 3D (C and D) CRISPR screens. Results of the Doxo-sensitive genes are displayed with blue headers (A and C), while the resistant ones are displayed with red headers (B and D). Each plot presents the enrichment score on the x-axis while KEGG pathway terms (top 10 significant) are displayed along the y-axis. The size and color of the dots corresponding to the gene count and p-values, respectively. The dashed boxes indicate the terms directly related to DNA damage-response.

In the 2D screens, Doxo-sensitive genes were enriched for cytoskeleton-related GO-BP terms, including intermediate filament cytoskeleton organization, intermediate filament-based processes, and apoptotic cell clearance (Fig. S2A). In contrast, 2D Doxo-resistant genes were primarily enriched for ribosome-related processes, such as rRNA processing, rRNA metabolic process, ribosome biogenesis, and ribosome assembly, as well as ncRNA-related processes including ncRNA metabolic process and ncRNA processing (Fig. S2B). KEGG pathway analysis revealed that 2D Doxo-sensitive genes were enriched for ABC transporters (hsa02010), Alcoholic liver disease (hsa04936), Glycosphingolipid biosynthesis (hsa00601), and Fatty acid biosynthesis (hsa00061) (Fig. 6A), whereas 2D Doxo-resistant genes were enriched for Nicotinate and nicotinamide metabolism (hsa00760), Basal transcription factors (hsa03022), and ABC transporters (hsa02010) (Fig. 6B).

**Fig. 7.**
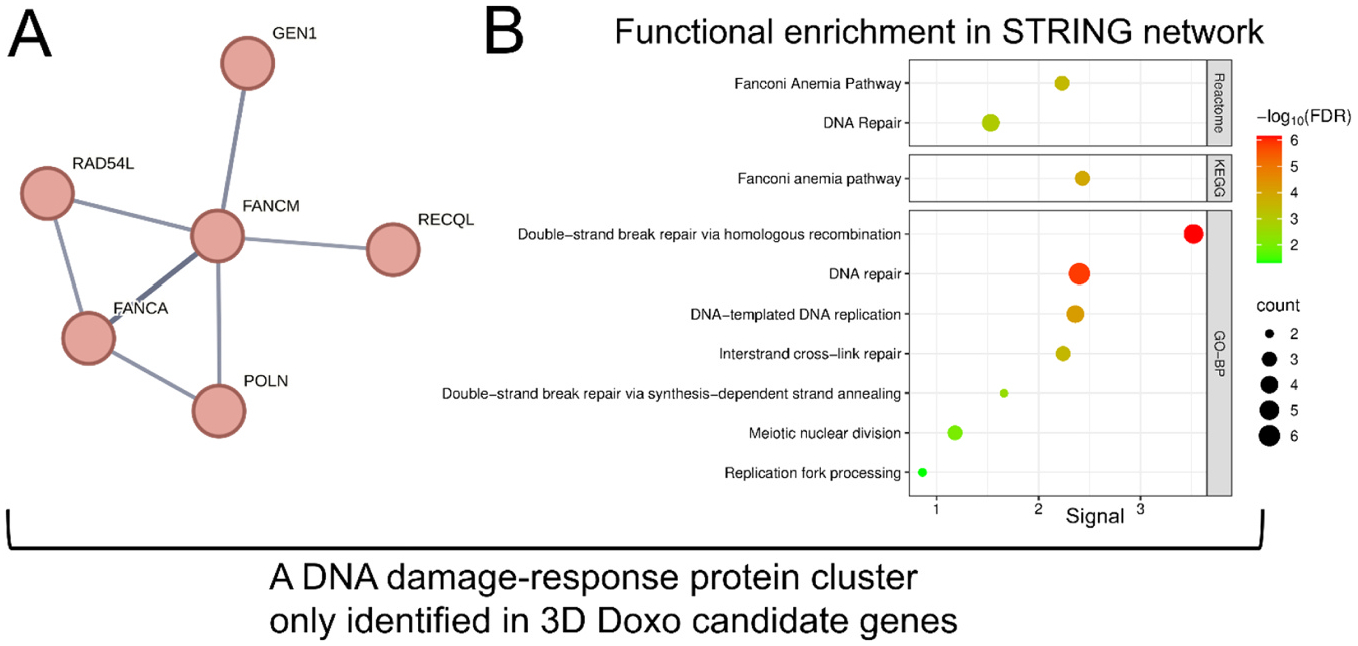
DNA damage response-related functional protein network significantly enriched in 3D doxorubicin (Doxo) candidate genes. (A) STRING protein network identified only in candidate genes of 3D Doxo CRISPR screens, not in the 2D screens. (B) Functional enrichment results reveal specific biological pathways related to DNA damage-response, including Reactome, KEGG, and GO-BP (Geno Ontology-Biological Process) terms. Each dot’s size and color correspond to the gene count and FDR statistical significance, respectively.

Given the substantially larger number of candidate modulators identified in the 3D screens, GO-BP and KEGG analyses captured more functional enrichment terms. For 3D Doxo-sensitive genes, the top GOBP term was store-operated calcium entry (GO:0002115) (Fig. S2C). The 3D Doxo-resistant genes were enriched for processes related to general toxicant response and metabolism, including xenobiotic metabolic process, cellular response to xenobiotic stimulus, epoxygenase P450 pathway, long-chain fatty acid metabolic process, and fatty acid metabolic process (Fig. S2D). KEGG pathway analysis of 3D Doxo candidate genes revealed pathways directly related to DNA-damage response, a known Doxo-induced toxicity mechanism(Pfitzer et al., 2019) (Figs. 6C and 6D). 3D Doxo-sensitive genes were enriched for Ascorbate and aldarate metabolism (hsa00053) as the top term, and included Chemical carcinogenesis–DNA adducts (hsa05204) and p53 signaling pathway (hsa04115), indicative of Doxo-associated mechanisms(Cutts et al., 2005; Poirier, 2012; Yimit et al., 2019; Shen et al., 2023) (Fig. 6C). Similarly, 3D Doxo-resistant genes showed enrichment of Chemical carcinogenesis–DNA adducts (hsa05204), and additional pathways associated with general toxicant metabolism, including Drug metabolism–cytochrome P450 (hsa00982), Retinol metabolism (hsa00830), ABC transporters (hsa02010), and PPAR signaling pathway (hsa03320) (Fig. 6D). STRING protein network analysis of all 3D Doxo candidate genes identified several functional clusters, including glycogen metabolism, xenobiotic metabolism (Fig. S3A and Fig. S3B), and DNA damage response–related pathways (Fig. 7), which were not captured in 2D screens. Notably, the DNA damage response cluster included *RAD54L*, *GEN1*, *RECQL*, *POLN*, *FANCM*, and *FANCA* (Fig. 7A) in the 3D screen. STRING functional enrichment revealed that this cluster was significantly associated with multiple Reactome, KEGG, and GO-BP terms, including Fanconi Anemia pathway (Reactome and KEGG), DNA repair (Reactome and GO-BP), Double-strand break repair via homologous recombination (GO-BP), DNA-templated DNA replication (GO-BP), Interstrand cross-link repair (GO-BP), Double-strand break repair via synthesis-dependent strand annealing (GO-BP), Meiotic nuclear division (GO-BP), and Replication fork processing (GO-BP) (Fig. 7B).

Altogether, our 3D CRISPR screen not only identified a greater number of candidate genetic modulators of Doxo-induced toxicity (enhanced discovery potential) but also captured chemical-specific biological pathways, demonstrating enhanced mechanistic resolution compared to the 2D system.

### 3.4. Candidate modulators of doxorubicin toxicity in 3D screen showed greater overlap with known doxorubicin phenotypes than modulators from the 2D screen

In our CRISPR screens, we used a custom sgRNA library representing the genes with the most common aggregate loss-of-function (LoF) mutations in the human population as of GnomAD V3.0 (Mean aggregate allele frequency of >0.1 %)(Kim et al., 2025). To evaluate the clinical relevance of candidate genetic modulators of cellular doxorubicin toxicity, we compared candidate genes identified in the 3D and 2D Doxo CRISPR screens with previously reported genetic variants with clinical phenotypes associated with doxorubicin treatment. We queried ClinPGx, a comprehensive pharmacogenomics resource that catalogs curated associations between genes (genetic variants) and drug response phenotype(Gong et al., 2025), for doxorubicin as well as daunorubicin, epirubicin, and idarubicin, which are considered to share common mechanism for inducing DNA damage, to capture a broad set of genetic variants associated with modulation of Doxo-related toxicity phenotypes.

**Fig. 8.**
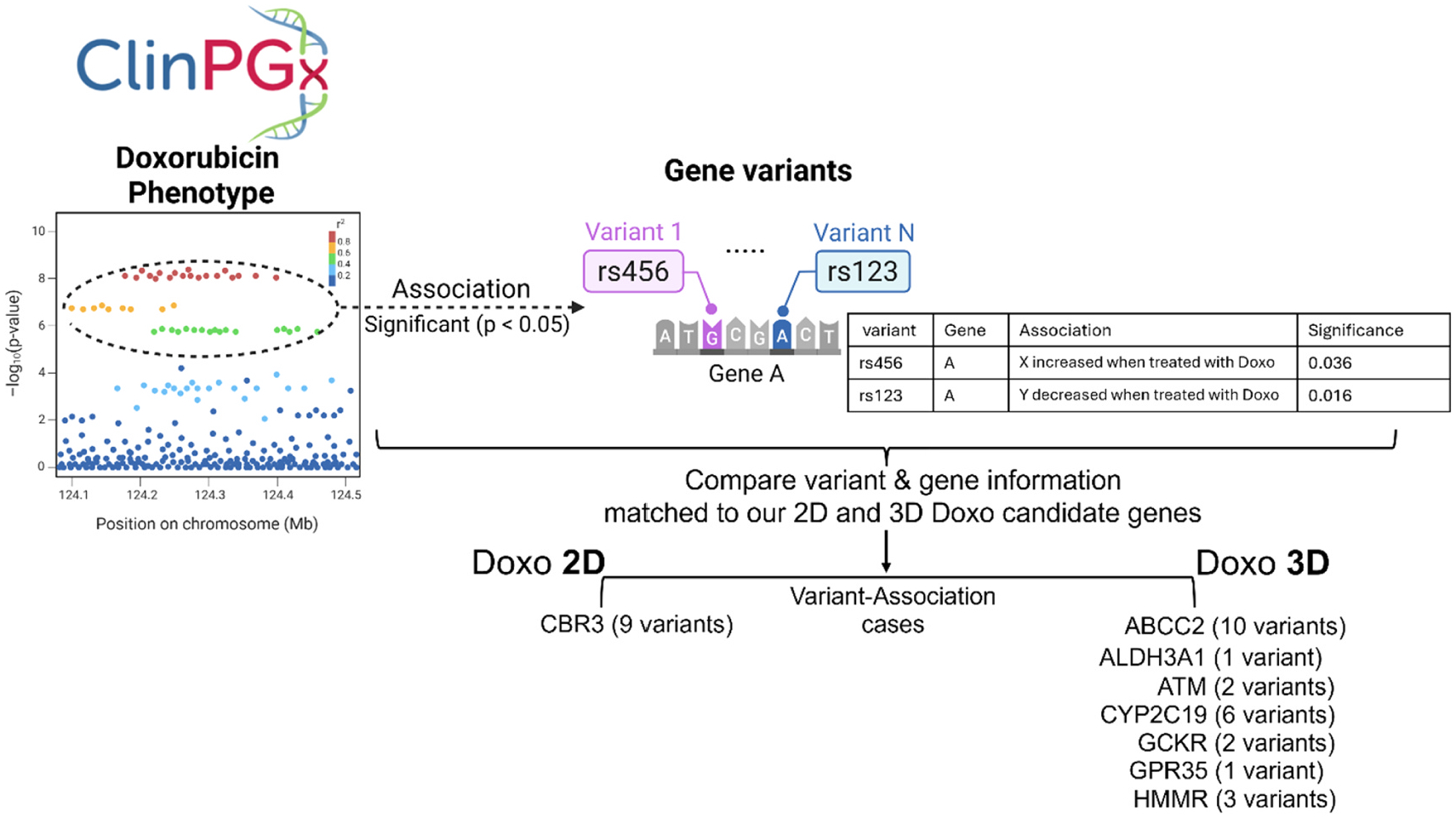
Comparison of candidate genetic modulators identified in 3D and 2D doxorubicin (Doxo) CRISPR screens with ClinPGx-reported gene–variant associations. Candidate genes identified in the 3D and 2D Doxo CRISPR screens were compared with genes and genetic variants previously associated with Doxo-related phenotypes using the ClinPGx database. The database was queried for four anthracycline drugs—doxorubicin, daunorubicin, epirubicin, and idarubicin—to capture a broad set of clinically relevant DNA damage–inducing phenotypes. Detailed information on individual variants, corresponding genes, associated phenotypes, and statistical significance values is provided in Table S4.

Among the 282 statistically significant gene–variant associations reported in ClinPGx, only *CBR3* (Table S4, Fig. 8) with 9 variant associations was also identified in the 2D CRISPR screen. In contrast, 7 of the 3D Doxo candidate genes identified in our study were also previously reported in ClinPGx. These include *ABCC2* (10 variant associations), *ALDH3A1* (1 variant association), *ATM* (2 variant associations), *CYP2C19* (6 variant associations), *GCKR* (2 variant associations), *GPR35* (1 variant association), and *HMMR* (3 variant associations) (Table S4, Fig. 8). These results demonstrate that candidate modulators identified in the 3D CRISPR screen exhibit stronger concordance with established gene–Doxo phenotype associations than those identified in the 2D screen, highlighting the potential enhanced functional relevance and predictive power of the 3D system. We provide the LoF frequency data (Mean Allele Frequency from gnomAD V3.0) of candidate genes identified in our chemical screening, including the Doxo phenotype-associated genes (Table S5).

## 4. Discussion

We established and applied a 3D CRISPR-based genetic screening platform to identify functional genetic determinants of chemical toxicity in a potentially more physiologically relevant culture context. Importantly, we focused on a limited subset of ~1550 human genes which contain common loss-of-function (LoF) mutations in the human population and thus, if functionally relevant to a specific toxicant exposure, could contribute to population relevant differences in human toxicity (Kim et al., 2025). Using doxorubicin as a model toxicant, we demonstrate that loss-of-function screening can be integrated with bioreactor-based 3D HepG2/C3A spheroids to resolve both shared and context-specific modulators of toxicant response. Building on our prior characterization of this spheroid system and its optimal exposure window (Kim et al., 2024), this study defines a robust workflow for comparative functional toxicogenomic analyses across 2D and 3D models. Collectively, our findings show that 3D screening captures distinct genetic pathways not fully recapitulated in 2D systems, underscoring the value of combining advanced 3D culture models with CRISPR-based functional genomics for next-generation NAM development.

We observed substantial differences in both the number and types of candidate genetic determinants influencing normal growth and chemical toxicity responses between 3D spheroid and 2D monolayer systems. We defined genes that, when disrupted, show increasing depletion over time, as time dependent essential (TDE) genes, while those genes showing increasing enrichment over time were classified as time dependent growth inhibition (TDGI) genes in each respective system. Our time-course CRISPR screens suggest unique and specific genetic components that are either disadvantageous or advantageous for comparative cell growth/survival in 3D and 2D. Notably, most genes that when targeted by CRISPR were comparatively less abundant in 2D culture have been reported to be essential(Wang et al., 2015) in the DepMap database(Lagziel et al., 2019), but most genes which were identified in the 3D CRISPR screen haven’t been documented as essential. This result not only supports the contention that our 2D time-course screen is comparable to previous 2D CRISPR screens which inform existing genetic essentiality data but also suggests that 3D culture time-course screening could provide new and physiologically relevant gene essentiality information. A growing body of evidence suggests that 3D cell culture systems, as compared to adherent 2D monoculture system, can more accurately represent the *in vivo* microenvironment in tissues(Edmondson et al., 2014; Law et al., 2021). The distinct cellular microenvironments, including access to nutrients, oxygen availability, and cellular state(Lagziel et al., 2019; Rossiter et al., 2021) may help explain the difference of functional effects of gene KO on cell growth/survival in the two systems. (Han et al., 2020; Grandhi et al., 2021).

Unexpectedly, we identified significantly more time dependent growth inhibition genes in 3D (355 genes) compared to 2D (4 genes). A STRING network analysis of these genes revealed functional enrichment in GO-CC terms associated with cell structure (Alberts et al., 2002; Manz and Groves, 2010; Huang et al., 2022). Since these structural components are required to maintain spheroid integrity, we hypothesize that these gene products may be necessary for constraining cellular proliferation in 3D, although the exact mechanisms remain to be determined (Heinrich et al., 2020; Carpenter et al., 2023). A previous study(Han et al., 2020) focused on cancer dependency also reported that a CRISPR screen identified more KOs with a growth advantage in 3D as compared to compared to the 2D screens, which is consistent with our findings.

We evaluated a 3D CRISPR screening platform relative to the 2D counterpart to identify genetic modulators and functional biological pathways underlying chemical-induced toxicity using Doxo as a model toxicant. We chose Doxo due to its well-characterized DNA-damaging mechanism: it intercalates into DNA and inhibits topoisomerases, blocking replication, transcription, and translation, ultimately inducing apoptosis in rapidly dividing cells (Thorn et al., 2011). Despite its broad chemotherapeutic use, Doxo is associated with severe side effects, including cardiotoxicity(Wang et al., 2009; Al-Otaibi et al., 2022; Rodrigues et al., 2022; Robert Li et al., 2024; Stafford et al., 2024; Suga et al., 2025) closely linked to its interactions with iron metabolism (Ye et al., 2024) which are believed to be distinct from the nuclear DNA damage mediated toxicity. Although less widely appreciated, hepatotoxicity(Prasanna et al., 2020) is a common side effect of anthracycline treatment. For instance, clinical data indicate that serum aminotransferase levels, such as alanine aminotransferase (ALT) and aspartate aminotransferase (AST), are elevated in up to 40 % of patients treated with Doxo, reflecting acute liver injury that is generally asymptomatic and transient. Hepatotoxicity remains a clinically relevant limitation (Prasanna et al., 2020; Lai et al., 2022) with its molecular mechanisms largely unexplored. Moreover, significant inter-individual variability in susceptibility to Doxo-induced hepatotoxicity and, cardiotoxicity suggests the presence of critical genetic determinants governing this adverse effect(Magdy et al., 2021; Xu et al., 2022; Fonoudi et al., 2024; El-Shorbagy et al., 2025; Zobeydi et al., 2025). We expected to find several common toxicity mechanisms and/or genes in response to Doxo in the contexts of cardio- and hepatotoxicity given that our screening approach is unbiased and systematic.

In our screens, multiple novel candidate genes were identified exclusively in the 3D system, but not in conventional 2D cultures. Notably, we detected significantly more Doxo-resistant genes in 3D (657 genes) compared to 2D (19 genes), whereas the difference for Doxo-sensitive genes was smaller (91 vs. 41 genes). The reason for this pronounced disparity in resistant genes remains unclear; however, one possible explanation is that the 3D spheroid environment more accurately recapitulates cellular physiology, producing a stronger selective effect of Doxo on cell survival and proliferation over the course of spheroid growth and chemical exposure with the similar cytotoxic effect (i.e., IC_25_)(Fey and Wrzesinski, 2012; Juarez-Moreno et al., 2022). These findings also have potential relevance to the development of chemotherapeutic resistance and suggest that 2D CRISPR screens may not identify clinically relevant resistance mechanisms that could be important in the 3D growth environment of solid tumors.

In addition to the difference in numbers of candidate genetic modulators of Doxo-induced toxicity identified in our screens, functional enrichment analysis across culture conditions demonstrated that the 3D CRISPR screen captured more candidates in biological pathways previously implicated in doxorubicin toxicity. Several pathways directly related to DNA damage-response were enriched exclusively in 3D screens, including chemical carcinogenesis-DNA adducts and p53 signaling pathway. Furthermore, STRING protein network analysis of the 3D candidate genetic modulators of Doxo-induced toxicity revealed a cluster linked to DNA damage-response pathways, such as Fanconi anemia (FA) pathway, DNA repair, and homologous recombination (HR). FA is a rare genomic instability disorder, often caused by mutations in genes that regulate replication-dependent removal of DNA crosslinks(Jenkins et al., 2012). The FA pathway coordinates multiple DNA repair mechanisms, leading to the formation of repair structures in response to genotoxic insults, such as Doxo(Moldovan and D’Andrea, 2009). In our analysis, both *FANCA* and *FANCM* were identified within the functionally enriched protein cluster among 3D Doxo candidate genes; these proteins form part of the FA core complex(Moldovan and D’Andrea, 2009; Jenkins et al., 2012). The HR process is closely associated with *RAD51* expression, and *RAD54L*—a member of the DNA damage–response functional cluster identified here—actively facilitates HR(Alagpulinsa et al., 2014). Collectively, these findings support the contention that 3D CRISPR screens, at least for Doxo, may identify chemical-specific mechanisms of toxicity more accurately than 2D screens, establishing 3D screening as an effective functional toxicogenomics approach. The enhanced performance of 3D CRISPR screens observed here is consistent with previous studies in cancer biology(Han et al., 2020; Takahashi et al., 2020). For instance, Han et al. reported that CRISPR phenotypes in 3D cancer spheroids more accurately recapitulate *in vivo* phenotypes than 2D systems, highlighting the improved physiological relevance of 3D screens for capturing cancer-related phenotypes(Han et al., 2020). Similarly, Takahashi et al. showed that 3D CRISPR screens more effectively reveal the molecular pathogenesis mediated by nuclear factor erythroid 2-related factor 2 (NRF2) hyperactivation in lung cancer compared to their 2D counterparts.(Takahashi et al., 2020).

Genome -wide association studies (GWAS) to identify genetic variants associated with differences in susceptibility to toxicants are challenging to carry out in human populations because of the complexity in human toxicant exposures and the requirement for large study populations(Simons et al., 2018; Genomics and Fletcher, 2023). However, some drug associated toxicities can be amendable to GWAS based approaches which can identify genetic variants associated with modulating adverse effects. Several GWAS studies of doxorubicin or related chemotherapeutics and targeted candidate gene association studies have identified genetic variants modulating susceptibility to anthracycline associated toxicity. A notable example is a GWAS in 280 European-ancestry patients (32 cases vs. 248 controls) with childhood cancer, which identified a non-synonymous variant (rs2229774, S427L) in *R*ARG as highly associated with Doxo-induced cardiotoxicity (Aminkeng et al., 2015). Gene-focused studies similarly highlighted the high interindividual variability in Doxo toxicity(Magdy et al., 2021), confirming the correlation of rs2229774 with adverse outcomes. We previously proposed that focusing on genes harboring common loss of function variants (PopVarLoF) in the human population may enable an alternative approach to GWAS or one by one candidate gene studies to identify population relevant variants impacting toxicant susceptibility (Kim et al., 2025). We thus compared our findings with the PopVarLoF set in the 2D and 3D screen to existing data gathered to date for Doxorubicin and related chemotherapeutics in the ClinPGx database, which compiles genetic variants associated with adverse phenotypes (WATANABE et al., 2023). Candidate genes modulating Doxo-induced toxicity in the 3D screen showed greater overlap with previously reported genes (7 genes: *ABCC2, ALDH3A1, ATM, CYP2C19, GCKR, GPR35,* and *HMMR*) with variants associated with adverse outcome differences than the one candidate gene, *CBR3,* identified in the 2D screen. The increased overlap between 3D screen candidates and ClinPGx-reported Doxo-associated genes (variants) supports the hypothesis that the 3D screening platform may better capture clinically relevant genetic modulators of Doxo cytotoxicity. At the same time, these screening studies alone cannot establish a clinical role nor provide definitive evidence of a functional role for any of the candidate genes identified in this study. Accordingly, these genes should be considered prioritized candidates for future targeted functional validation and clinical association studies and may represent false-positives. The identification of *CBR3* as a sensitivity gene (in our 2D screens) of Doxo-induced toxicity is aligned with the functional impact of *CBR3* KO, experimentally validated in a previous study(Fonoudi et al., 2024). In addition, *CYP2C19* identified as Doxo-resistant gene in our 3D CRISPR screens is consistent with the reported functional role of a *CYP2C19* variant (rs4244285) in production of nonfunctional enzyme (i.e., LoF), which may reduce cellular metabolic capacity of Doxo, resulting in attenuated toxicity. Although we used the common LoF mutation genes for sgRNA library which might limit the capacity of identification of genes modulating Doxo-induced toxicity, these findings support that our approach and screening system are reliable. Given that association-based evidence alone cannot establish causal relationships, the identified genes should be considered prioritized candidates for future targeted functional validation, and we acknowledge the possibility of false-positive hits. We found additional candidates that could be relevant to Doxo phenotypes that had not been previously identified in candidate gene or genome wide association studies. For example, we identified multiple *UGT2A* and *UGT2B* isoforms involved in glucuronidation, including *UGT2A1* (sensitive), *UGT2A2* (resistant), *UGT2B10* (sensitive), and *UGT2B11* (resistant), which were significantly differentially selected (Table S4). Polymorphisms in a related gene *UGT2B7* have been previously shown to reduce glucuronidation activity, reduce clearance, leading to increased systemic exposure to Doxo and a higher risk of liver injury(Innocenti et al., 2001).(Hu et al., 2014, 2015). The identification of these new *UGT2A/B* candidate may reflect a role for these isoforms in the glucuronidation of different doxorubicin metabolites or potential changes in the extracellular matrix as suggested previously(Vitale et al., 2024). Notably, the entire set of 3D Doxo-sensitive genes was enriched for the pentose and glucuronate interconversions pathway (Fig. 6C), which is directly involved in glucuronidation(Kato et al., 2013; Oda et al., 2015). These findings suggest that follow-up investigations into functional or causal genetic variation-harboring genes identified in our screens (Table S4), which were preselected for population relevant genes with common LoF mutations, could improve the identification of genetic factors (e.g. predictive biomarkers) contributing to Doxo-induced liver toxicity that might have been missed by conventional GWAS due to limited patient numbers, stringent statistical thresholds, or lack of functional validation(Wang et al., 2016; Schneider et al., 2017; Wells et al., 2017).

## 5. Limitations of the study

The study focuses on a subset of genes in which loss of function alleles are common in human population and thus will not identify genes with less common LoF alleles, including genes previously identified to play a role in Doxo-induced toxicity, and similarly does not include other potential alleles, such as gain of function, which could influence the toxicity. Our study did not carry out single-gene validation of each candidate gene in line with our goal to compare gene- and pathway-level responses to Doxo across 2D and 3D culture systems. Future studies incorporating targeted genetic perturbation and mechanistic validation will be required to confirm the functional roles of these candidates in the contexts of both Doxo-induced hepatotoxicity in 2D vs. 3D cultures, as well as any role in clinical toxicity observed in anthracycline treatment in people.

We recognize that the difference in numbers and identities of gene-level hits of 2D vs. 3D screening platforms could be derived from experimental noise sources so we did not rely on single-gene overlaps but emphasize the systematic pattern of each condition (e.g., pathways). Moreover, our experimental workflow is robust in that we used the same sgRNA library, viral batch, MOI, selection strategy, sequencing platform, and computational pipeline which allows us to contend that the primary experimental variable is the difference in cell culture systems. Moreover, pooled CRISPR screens inherently incorporate substantial internal replication, as each sgRNA is sampled across hundreds of thousands of cells under high library coverage. The use of extensive negative controls enables robust variance estimation and reduces sgRNA-specific artifacts. We also acknowledge that additional independent experimental replicates can provide support for a more robustness of the identified hits. However, our goal was to demonstrate the capacity of the 3D CRISPR screening system to uncover culture system-specific genetic dependencies in time-course and chemical toxicity contexts.

## 6. Conclusion

Currently available genetic approaches to address population variability and chemical susceptibility often lack diversity in study populations, limiting their ability to capture the full spectrum of variability. They also frequently face challenges in establishing causative relationships due to the inherent limitations of correlation-based methods(Amos et al., 2011). Our integrative CRISPR-based approach addresses these gaps by enabling prediction, prevention, and mechanistic understanding of Doxo-induced toxicity, as well as other liver-specific adverse outcomes or diseases. This approach bridges GWAS and gene-centric strategies by providing an intermediate, scalable method to identify population relevant functional genetic determinants that could influence response to toxicants, overcoming the limitations of each. More broadly, the ability to systematically identify common genetic components—aggregate variants required for survival under distinct growth conditions, in the absence or presence of a toxicant—can enhance our understanding of functional genetic elements, context-specific susceptibility, regulatory networks, and toxicant-specific modulators across different culture systems.

In this study, we demonstrate that a 3D spheroid-based CRISPR screening platform identifies distinct genetic determinants of chemical-induced toxicity compared to conventional 2D monolayer screens. Time-course CRISPR screens under basal conditions revealed that 3D cultures identify markedly different patterns of genes modulating both relative growth/survival as compared to 2D cultures. These differences highlight that cellular architecture, microenvironment, and metabolic state likely alter genetic dependencies, underscoring the importance of physiologically relevant systems for functional genomics.

When applied to doxorubicin, a DNA-damaging toxicant, the 3D CRISPR screen identified more candidate genetic modulators than the 2D screen and uniquely captured pathways consistent with established Doxo toxicity mechanisms—including Fanconi anemia signaling, double-strand break repair, replication fork processing pathways. In contrast, the 2D system predominantly recovered general stress-related processes rather than chemical-specific mechanisms. Importantly, candidate genes identified in 3D showed substantially greater overlap with known clinical pharmacogenomic associations for anthracycline toxicity, demonstrating improved alignment with human susceptibility and validating the translational relevance of the 3D platform.

Collectively, these findings show that 3D spheroid-based CRISPR screening markedly enhances the discovery of mechanistically meaningful gene–toxicant interactions and improves the detection of inter-individual susceptibility factors compared to 2D approaches. Our results support the use of 3D genetic screening as a next-generation New Approach Methodology (NAM) for mechanistic toxicology and chemical safety evaluation. This platform offers a powerful strategy for defining causal genetic contributors to chemical responses, improving prediction of human-relevant toxicity pathways, and advancing the development of more accurate and physiologically grounded toxicological models.

## Supplementary Material

Supplemental Material

Supplementary material is available at *Toxicology* online.

## Figures and Tables

**Table 1:** Comparison of time-dependent essential (TDE) genes (growth advantage genes) identified in time-course CRISPR screens in 2D monolayer and 3D spheroid systems with the gene essentiality profile catalogued in DepMap database(Tsherniak et al., 2017). Continuous depletion of a gene KO mutant was empirically determined based on decreasing Log_2_FC values over time (Day 20 to Day 30) and gene KO mutants with such patterns were identified as TDE genes in the screens. Gene effect score less than 0 indicates gene essentiality where a score near − 1 suggests that the gene is strongly essential (based on the Chronos dependency score from DepMap; DepMap: The Cancer Dependency Map Project at Broad Institute)(Tsherniak et al., 2017).

	TDE GENE	Day20_Log_2_FC Vs. Day 0 (Empirical)	Day30_Log_2_FC Vs. Day 0 (Empirical)	Gene Effect (DepMap)	Gene Essentiality (DepMap)
**2D**	*ABCB7*	−2.86	−3.15	−1.90	Essential
	*ADAM2*	−1.77	−1.88	0.001	Not essential
	*DDX11*	−1.32	−2.04	−0.67	Essential
	*DDX52*	−1.51	−2.39	−0.47	Essential
	*DIS3*	−1.87	−2.09	−0.64	Essential
	*MYBBP1A*	−2.95	−3.11	−0.46	Essential
	*POLR3C*	−1.71	−2.38	−1.14	Essential
	*YARS2*	−3.22	−3.75	−1.02	Essential
	*GOLGA8S*	−2.58	−3.14	No data	No data
**3D**	*CCDC63*	−2.91	−3.15	−0.21	Essential
	*FDXACB1*	−3.17	−3.51	0.01	Not essential
	*MYH7B*	−3.10	−3.62	No data	No data
	*NBPF9*	−3.43	−3.73	No data	No data
	*TAF6*	−2.74	−3.80	−1.65	Essential
	*TLP1*	−2.18	−3.25	No data	No data

**Table 2:** Comparison of time-dependent growth inhibition (TDGI) genes identified in time-course CRISPR screens in 2D monolayer and 3D spheroid systems. Continuous enrichment of a gene KO mutant was empirically determined based on increasing Log_2_FC values over time (Day 20 to Day 30) and gene KOs with such pattern were identified as TDGI in the screens. The entire list of 386 nTDGI genes identified in the 3D system is available in Supplementary Table S3.

	TDGI GENE	Day20_Log_2_FC Vs. Day 0 (Empirical)	Day30_Log_2_FC Vs. Day 0 (Empirical)
**2D**	*CYP2A13*	1.21	1.31
	*KMT2C*	1.08	1.08
	*OTOP3*	1.21	1.53
	*ZNF556*	1.5	1.54
**3D**	*A2ML1*	2.39	3.79
	*ABCA10*	1.99	4.40
	*ABCB5*	2.86	5.11
	…	…	…
	*UGT1A10*	3.51	6.75
	*UGT2A1*	2.82	5.90

## Data Availability

Data will be made available on request.

## Appendix A. Supporting information

Supplementary data associated with this article can be found in the online version at doi:10.1016/j.tox.2026.154422.

## References

[R1] Alagpulinsa DA, Ayyadevara S, Shmookler Reis RJ, 2014. A Small-Molecule Inhibitor of RAD51 Reduces Homologous Recombination and Sensitizes Multiple Myeloma Cells to Doxorubicin. Front Oncol. 4. 10.3389/fonc.2014.00289. PMC4214226 25401086

[R2] Alberts B, Johnson A, Lewis J, , 2002. Membrane Proteins. Molecular Biology of the Cell, 4th edition. Garland Science.

[R3] Alexander-Dann B, Pruteanu LL, Oerton E, , 2018. Developments in toxicogenomics: understanding and predicting compound-induced toxicity from gene expression data. Mol. Omics 14, 218–236. 10.1039/c8mo00042e. 29917034 PMC6080592

[R4] Al-Otaibi TK, Weitzman B, Tahir UA, , 2022. Genetics of Anthracycline-Associated Cardiotoxicity. Front Cardiovasc Med 9, 867873. 10.3389/fcvm.2022.867873. 35528837 PMC9068960

[R5] Aminkeng F, Bhavsar AP, Visscher H, , 2015. A coding variant in RARG confers susceptibility to anthracycline-induced cardiotoxicity in childhood cancer. Nat. Genet 47, 1079–1084. 10.1038/ng.3374. 26237429 PMC4552570

[R6] Amos W, Driscoll E, Hoffman JI, 2011. Candidate genes versus genome-wide associations: which are better for detecting genetic susceptibility to infectious disease? Proc. Biol. Sci 278, 1183–1188. 10.1098/rspb.2010.1920. 20926441 PMC3049081

[R7] Anon. Recent advances in 2D and 3D in vitro systems using primary hepatocytes, alternative hepatocyte sources and non-parenchymal liver cells and their use in investigating mechanisms of hepatotoxicity, cell signaling and ADME | SpringerLink. Available at: 〈https://link.springer.com/article/10.1007/s00204-013-1078-5〉 [Accessed August 10, 2023]. 10.1007/s00204-013-1078-5 PMC3753504 23974980

[R8] Arroyo JD, Jourdain AA, Calvo SE, , 2016. A Genome-wide CRISPR Death Screen Identifies Genes Essential for Oxidative Phosphorylation. Cell Metab. 24, 875–885. 10.1016/j.cmet.2016.08.017. 27667664 PMC5474757

[R9] Asmamaw M, Zawdie B, 2021. Mechanism and Applications of CRISPR/Cas-9- Mediated Genome Editing. Biol. Targets Ther 15, 353. 10.2147/BTT.S326422. PMC8388126 34456559

[R10] Awah CU, Winter J, Ogunwobi OO, 2022. Genome scale CRISPR Cas9a knockout screen reveals genes that control glioblastoma susceptibility to the alkylating agent temozolomide. All Life 15, 88–93. 10.1080/26895293.2021.2024895. 35990011 PMC9389140

[R11] Barrangou R, Doudna JA, 2016. Applications of CRISPR technologies in research and beyond. Nat. Biotechnol 34, 933–941. 10.1038/nbt.3659. 27606440

[R12] Beale DJ, Sinclair GM, Shah R, , 2022. A review of omics-based PFAS exposure studies reveals common biochemical response pathways. Sci. Total Environ 845, 157255. 10.1016/j.scitotenv.2022.157255. 35817100

[R13] Beilmann M, Adkins K, Boonen HCM, , 2025. Application of new approach methodologies for nonclinical safety assessment of drug candidates. Nat. Rev. Drug Discov 1–21. 10.1038/s41573-025-01182-9. 40316753

[R14] Bock C, Datlinger P, Chardon F, , 2022. High-content CRISPR screening. Nat. Rev. Methods Prim 2, 1–23. 10.1038/s43586-021-00093-4. PMC10200264 37214176

[R15] Carpenter LC, Pérez-Verdugo F, Banerjee S, 2023. Mechanical control of cell proliferation patterns in growing tissues. bioRxiv, 2023.07.25.550581. 10.1101/2023.07.25.550581. PMC10995431 38449309

[R16] Chang J, Wang R, Yu K, , 2020. Genome-wide CRISPR screening reveals genes essential for cell viability and resistance to abiotic and biotic stresses in Bombyx mori. Genome Res 30, 757–767. 10.1101/gr.249045.119. 32424075 PMC7263191

[R17] Chen C-H, Xiao T, Xu H, , 2018. Improved design and analysis of CRISPR knockout screens. Bioinformatics 34, 4095–4101. 10.1093/bioinformatics/bty450. 29868757 PMC6247926

[R18] Chen X, Lu N, Huang S, , 2023. Assessment of doxorubicin toxicity using human cardiac organoids: A novel model for evaluating drug cardiotoxicity. Chem. Biol. Interact 386, 110777. 10.1016/j.cbi.2023.110777. 37879593

[R19] Cutts SM, Nudelman A, Rephaeli A, , 2005. The power and potential of doxorubicin-DNA adducts. IUBMB Life 57, 73–81. 10.1080/15216540500079093. 16036566

[R20] Doench JG, 2018. Am I ready for CRISPR? A user’s guide to genetic screens. Nat. Rev. Genet 19, 67–80. 10.1038/nrg.2017.97. 29199283

[R21] Duval K, Grover H, Han L-H, , 2017. Modeling Physiological Events in 2D vs. 3D Cell Culture. Physiol. (Bethesda) 32, 266–277. 10.1152/physiol.00036.2016. PMC5545611 28615311

[R22] Edmondson R, Broglie JJ, Adcock AF, , 2014. Three-Dimensional Cell Culture Systems and Their Applications in Drug Discovery and Cell-Based Biosensors. ASSAY Drug Dev. Technol 12, 207–218. 10.1089/adt.2014.573. 24831787 PMC4026212

[R23] El-Shorbagy EA, El-Bassiouny NA, Kassem AB, , 2025. The impact of CBR3 (rs1056892) and ABCC1 (rs45511401) genetic polymorphisms on doxorubicin-induced cardiotoxicity and the potential role of brain natriuretic peptide as an early cardiac biomarker in breast cancer patients. Expert Opin. Drug Metab. Toxicol 1–13. 10.1080/17425255.2025.2490736. 40207889

[R24] Fey SJ, Wrzesinski K, 2012. Determination of Drug Toxicity Using 3D Spheroids Constructed From an Immortal Human Hepatocyte Cell Line. Toxicol. Sci 127, 403–411. 10.1093/toxsci/kfs122. 22454432 PMC3355318

[R25] Fonoudi H, Jouni M, Cejas RB, , 2024. Functional Validation of Doxorubicin-Induced Cardiotoxicity-Related Genes. JACC CardioOncology 6, 38–50. 10.1016/j.jaccao.2023.11.008. 38510289 PMC10950437

[R26] Gaytán BD, Vulpe CD, 2014. Functional toxicology: tools to advance the future of toxicity testing. Front Genet 5, 110. 10.3389/fgene.2014.00110. 24847352 PMC4017141

[R27] Genomics FL and Fletcher L (2023). How-to: perform a genome-wide association study (GWAS). Front Line Genomics. Available at: 〈https://frontlinegenomics.com/how-to-perform-a-genome-wide-association-study-gwas/〉 [Accessed July 29, 2024].

[R28] Gong L, Klein CJ, Caudle KE, , 2025. Integrating Pharmacogenomics into the Broader Construct of Genomic Medicine: Efforts by the ClinGen Pharmacogenomics Working Group (PGxWG). Clin. Chem 71, 36–44. 10.1093/clinchem/hvae181. 39749515 PMC12037359

[R29] Grandhi TSP, To J, Romero A, , 2021. High-throughput CRISPR-mediated 3D enrichment platform for functional interrogation of chemotherapeutic resistance. Biotechnol. Bioeng 118, 3187–3199. 10.1002/bit.27844. 34050941

[R30] Gunness P, Mueller D, Shevchenko V, , 2013. 3D Organotypic cultures of human heparg cells: a tool for in vitro toxicity studies. Toxicol. Sci 133, 67–78. 10.1093/toxsci/kft021. 23377618

[R31] Han K, Pierce SE, Li A, , 2020. CRISPR screens in cancer spheroids identify 3D growth-specific vulnerabilities. Nature 580, 136–141. 10.1038/s41586-020-2099-x. 32238925 PMC7368463

[R32] Harrill JA, Everett LJ, Haggard DE, , 2021. High-Throughput Transcriptomics Platform for Screening Environmental Chemicals. Toxicol. Sci 181, 68–89. 10.1093/toxsci/kfab009. 33538836 PMC10194851

[R33] Hasin Y, Seldin M, Lusis A, 2017. Multi-omics approaches to disease. Genome Biol. 18, 83. 10.1186/s13059-017-1215-1. 28476144 PMC5418815

[R34] Heinrich MA, Alert R, LaChance JM, , 2020. Size-dependent patterns of cell proliferation and migration in freely-expanding epithelia. In: Rosenblatt J, Stainier DY, Kabla A (Eds.), eLife 9, e58945. 10.7554/eLife.58945. 32812871 PMC7498264

[R35] Hu DG, Mackenzie PI, Lu L, , 2015. Induction of human UDP-Glucuronosyltransferase 2B7 gene expression by cytotoxic anticancer drugs in liver cancer HepG2 cells. Drug Metab. Dispos 43, 660–668. 10.1124/dmd.114.062380. 25713207

[R36] Hu DG, Rogers A, Mackenzie PI, 2014. Epirubicin upregulates UDP glucuronosyltransferase 2B7 expression in liver cancer cells via the p53 pathway. Mol. Pharm 85, 887–897. 10.1124/mol.114.091603. 24682467

[R37] Huang WYC, Cheng X, Ferrell JE, 2022. Cytoplasmic organization modulates reaction kinetics in cells. Biophys. J 121, 149a. 10.1016/j.bpj.2021.11.1970.

[R38] Innocenti F, Iyer L, Ramírez J, , 2001. Epirubicin Glucuronidation Is Catalyzed by Human UDP-Glucuronosyltransferase 2B7. Drug Metab. Dispos 29, 686–692. 10.1124/dmd.29.5.686. 11302935

[R39] Jenkins C, Kan J, Hoatlin ME, 2012. Targeting the Fanconi Anemia Pathway to Identify Tailored Anticancer Therapeutics. Anemia 2012, 481583. 10.1155/2012/481583. 22693661 PMC3368156

[R40] Jinek M, Chylinski K, Fonfara I, , 2012. A programmable dual-RNA-guided DNA endonuclease in adaptive bacterial immunity. Science 337, 816–821. 10.1126/science.1225829. 22745249 PMC6286148

[R41] Juarez-Moreno K, Chávez-García D, Hirata G, , 2022. Monolayer (2D) or spheroids (3D) cell cultures for nanotoxicological studies? Comparison of cytotoxicity and cell internalization of nanoparticles. Toxicol. Vitr 85, 105461. 10.1016/j.tiv.2022.105461. 36049398

[R42] Kapałczyńska M, Kolenda T, Przybyła W, , 2018. 2D and 3D cell cultures – a comparison of different types of cancer cell cultures. Arch. Med Sci 14 910919. 10.5114/aoms.2016.63743. PMC6040128 30002710

[R43] Karczewski KJ, Francioli LC, Tiao G, , 2020. The mutational constraint spectrum quantified from variation in 141,456 humans. Nature 581, 434–443. 10.1038/s41586-020-2308-7. 32461654 PMC7334197

[R44] Kato Y, Izukawa T, Oda S, , 2013. Human UDP-Glucuronosyltransferase (UGT) 2B10 in Drug N-glucuronidation: substrate screening and comparison with UGT1A3 and UGT1A4. Drug Metab. Dispos 41, 1389–1397. 10.1124/dmd.113.051565. 23611809

[R45] Kim C, Zhu Z, Barbazuk WB, , 2024. Time-course characterization of whole-transcriptome dynamics of HepG2/C3A spheroids and its toxicological implications. Toxicol. Lett 401, 125–138. 10.1016/j.toxlet.2024.10.004. 39368564 PMC12087462

[R46] Kim C, Zhu Z, Tagmount A (2025). Characterizing common loss-of-function genes and their potential utility in assessing population variability and chemical susceptibility, 2025.12.16.694775. 10.64898/2025.12.16.694775.

[R47] Lagziel S, Lee WD, Shlomi T, 2019. Inferring cancer dependencies on metabolic genes from large-scale genetic screens. BMC Biol. 17, 37. 10.1186/s12915-019-0654-4. 31039782 PMC6489231

[R48] Lai C, Cole DE, Steinberg SM, , 2022. Doxorubicin pharmacokinetics and toxicity in patients with aggressive lymphoma and hepatic impairment. Blood Adv. 7, 529–532. 10.1182/bloodadvances.2022007431. PMC9979767 35882475

[R49] Law AMK, Rodriguez de la Fuente L, Grundy TJ, , 2021. Advancements in 3D Cell Culture Systems for Personalizing Anti-Cancer Therapies. Front Oncol. 11, 782766. 10.3389/fonc.2021.782766. 34917509 PMC8669727

[R50] Li W, Xu H, Xiao T, , 2014. MAGeCK enables robust identification of essential genes from genome-scale CRISPR/Cas9 knockout screens. Genome Biol. 15, 554. 10.1186/s13059-014-0554-4. 25476604 PMC4290824

[R51] Love MI, Huber W, Anders S, 2014. Moderated estimation of fold change and dispersion for RNA-seq data with DESeq2. Genome Biol. 15, 550. 10.1186/s13059-014-0550-8. 25516281 PMC4302049

[R52] Magdy T, Jiang Z, Jouni M, , 2021. RARG variant predictive of doxorubicin-induced cardiotoxicity identifies a cardioprotective therapy. Cell Stem Cell 28, 2076–2089.e7. 10.1016/j.stem.2021.08.006. 34525346 PMC8642268

[R53] Manz BN, Groves JT, 2010. Spatial organization and signal transduction at intercellular junctions. Nat. Rev. Mol. Cell Biol 11, 342–352. 10.1038/nrm2883. 20354536 PMC3693730

[R54] Moldovan G-L, D’Andrea AD, 2009. How the Fanconi Anemia pathway guards the genome. Annu Rev. Genet 43, 223–249. 10.1146/annurev-genet-102108-134222. 19686080 PMC2830711

[R55] Morgens DW, Wainberg M, Boyle EA, , 2017. Genome-scale measurement of off-target activity using Cas9 toxicity in high-throughput screens. Nat. Commun 8, 15178. 10.1038/ncomms15178. 28474669 PMC5424143

[R56] Nicoletto RE, Ofner CM, 2022. Cytotoxic mechanisms of doxorubicin at clinically relevant concentrations in breast cancer cells. Cancer Chemother. Pharm 89, 285–311. 10.1007/s00280-022-04400-y. 35150291

[R57] Oda S, Fukami T, Yokoi T, , 2015. A comprehensive review of UDP-glucuronosyltransferase and esterases for drug development. Drug Metab. Pharmacokinet 30, 30–51. 10.1016/j.dmpk.2014.12.001. 25760529

[R58] Pfitzer L, Moser C, Gegenfurtner F, , 2019. Targeting actin inhibits repair of doxorubicin-induced DNA damage: a novel therapeutic approach for combination therapy. Cell Death Dis. 10, 302. 10.1038/s41419-019-1546-9. 30944311 PMC6447524

[R59] Poirier MC, 2012. Chemical-induced DNA Damage and Human Cancer Risk. Discov. Med 14, 283–288. 23114584 PMC7493822

[R60] Prasanna PL, Renu K, Valsala Gopalakrishnan A, 2020. New molecular and biochemical insights of doxorubicin-induced hepatotoxicity. Life Sci. 250, 117599. 10.1016/j.lfs.2020.117599. 32234491

[R61] Robert Li Y, Traore K, Zhu H, 2024. Novel molecular mechanisms of doxorubicin cardiotoxicity: latest leading-edge advances and clinical implications. Mol. Cell Biochem 479, 1121–1132. 10.1007/s11010-023-04783-3. 37310587

[R62] Rodrigues D, Coyle L, Füzi B, , 2022. Unravelling mechanisms of doxorubicin-induced toxicity in 3d human intestinal organoids. Int. J. Mol. Sci 23, 1286. 10.3390/ijms23031286. 35163210 PMC8836276

[R63] Rossiter NJ, Huggler KS, Adelmann CH, , 2021. CRISPR screens in physiologic medium reveal conditionally essential genes in human cells. Cell Metab. 33, 1248–1263.e9. 10.1016/j.cmet.2021.02.005. 33651980 PMC8172426

[R64] Schneider BP, Shen F, Gardner L, , 2017. Genome-Wide Association Study for Anthracycline-Induced Congestive Heart Failure. Clin. Cancer Res 23, 43–51. 10.1158/1078-0432.CCR-16-0908. 27993963 PMC5215621

[R65] Shen J, Wang Q, Mao Y, , 2023. Targeting the p53 signaling pathway in cancers: Molecular mechanisms and clinical studies. MedComm 4, e288. 10.1002/mco2.288. 37256211 PMC10225743

[R66] Simons YB, Bullaughey K, Hudson RR, , 2018. A population genetic interpretation of GWAS findings for human quantitative traits. PLOS Biol. 16, e2002985. 10.1371/journal.pbio.2002985. 29547617 PMC5871013

[R67] Sobh A, Loguinov A, Stornetta A, , 2019. Genome-Wide CRISPR Screening Identifies the Tumor Suppressor Candidate OVCA2 As a Determinant of Tolerance to Acetaldehyde. Toxicol. Sci 169, 235–245. 10.1093/toxsci/kfz037. 31059574 PMC6484886

[R68] Sobh A, Loguinov A, Yazici GN, , 2019. Functional Profiling Identifies Determinants of Arsenic Trioxide Cellular Toxicity. Toxicol. Sci 169, 108–121. 10.1093/toxsci/kfz024. 30815697 PMC6484884

[R69] Sobh A, Russo M, Vulpe CD, 2021. CRISPR Screens in Toxicology Research: An Overview. Curr. Protoc 1, e136. 10.1002/cpz1.136. 34043288

[R70] Sobh A, Vulpe C, 2019. CRISPR genomic screening informs gene–environment interactions. Curr. Opin. Toxicol 18, 46–53. 10.1016/j.cotox.2019.02.009.

[R71] Stafford LK, Tang X, Brandt A, , 2024. Risk of anthracycline-induced cardiac dysfunction in adolescent and young adult (AYA) cancer survivors: role of genetic susceptibility loci. Pharm. J 24, 1–7. 10.1038/s41397-024-00343-0. PMC11824427 38951505

[R72] Stresser DM, Kopec AK, Hewitt P, , 2024. Towards in vitro models for reducing or replacing the use of animals in drug testing. Nat. Biomed. Eng 8, 930–935. 10.1038/s41551-023-01154-7. 38151640

[R73] Suga T, Kitani T, Kogure M, , 2025. Thousand and one amino acid protein kinase 1 suppression improves doxorubicin-induced cardiomyopathy by preventing cardiomyocyte death and dysfunction. Cardiovasc. Res, cvaf022 10.1093/cvr/cvaf022. 39964965

[R74] Szklarczyk D, Kirsch R, Koutrouli M, , 2023. The STRING database in 2023: protein-protein association networks and functional enrichment analyses for any sequenced genome of interest. Nucleic Acids Res 51, D638–D646. 10.1093/nar/gkac1000. 36370105 PMC9825434

[R75] Takahashi N, Cho P, Selfors LM, , 2020. 3D Culture Models with CRISPR Screens Reveal Hyperactive NRF2 as a prerequisite for spheroid formation via regulation of proliferation and ferroptosis. Mol. Cell 80, 828–844.e6. 10.1016/j.molcel.2020.10.010. 33128871 PMC7718371

[R76] Tang D, Chen M, Huang X, , 2023. SRplot: a free online platform for data visualization and graphing. PLOS ONE 18, e0294236. 10.1371/journal.pone.0294236. 37943830 PMC10635526

[R77] Tang J, Shi J, Liu J, 2023. Editorial: Advances in 3D cell culture for drug screening and toxicology evaluation. Front Bioeng. Biotechnol 11, 1266506. 10.3389/fbioe.2023.1266506. 37593324 PMC10431958

[R78] Thorn CF, Oshiro C, Marsh S, , 2011. Doxorubicin pathways: pharmacodynamics and adverse effects. Pharm. Genom 21, 440. 10.1097/FPC.0b013e32833ffb56. PMC3116111 21048526

[R79] Tsherniak A, Vazquez F, Montgomery PG, , 2017. Defining a Cancer Dependency Map. Cell 170, 564–576.e16. 10.1016/j.cell.2017.06.010. 28753430 PMC5667678

[R80] Vitale DL, Parnigoni A, Viola M, , 2024. Deciphering drug resistance: investigating the emerging role of hyaluronan metabolism and signaling and tumor extracellular matrix in cancer chemotherapy. Int J. Mol. Sci 25, 7607. 10.3390/ijms25147607. 39062846 PMC11276752

[R81] Wang B, Chen JZ, Luo XQ, , 2022. The application of genome-wide CRISPR-Cas9 screens to dissect the molecular mechanisms of toxins. Comput. Struct. Biotechnol. J 20, 5076–5084. 10.1016/j.csbj.2022.09.012. 36187925 PMC9489804

[R82] Wang J, Reijmers T, Chen L, , 2009. Systems toxicology study of doxorubicin on rats using ultra performance liquid chromatography coupled with mass spectrometry based metabolomics. Metabolomics 5, 407–418. 10.1007/s11306-009-0165-3. 20046867 PMC2794350

[R83] Wang T, Birsoy K, Hughes NW, , 2015. Identification and characterization of essential genes in the human genome. Science 350, 1096–1101. 10.1126/science.aac7041. 26472758 PMC4662922

[R84] Wang X, Sun C-L, Quiñones-Lombraña A, , 2016. CELF4 Variant and anthracycline-related cardiomyopathy: a children’s oncology group genome-wide association study. J. Clin. Oncol 34, 863–870. 10.1200/JCO.2015.63.4550. 26811534 PMC5070560

[R85] WATANABE M, KAKUTANI M, ANDO R, , 2023. Susceptibility to adriamycin-induced hepatotoxicity in mice depends on PRKDC polymorphism. J. Vet. Med Sci 85, 702–704. 10.1292/jvms.23-0115. 37245991 PMC10372250

[R86] Wells QS, Veatch OJ, Fessel JP, , 2017. Genome-wide association and pathway analysis of left ventricular function after anthracycline exposure in adults. Pharm. Genom 27, 247–254. 10.1097/FPC.0000000000000284. PMC5502740 28542097

[R87] Xu X, Liu Q, Li J, , 2022. Co-Treatment With Resveratrol and FGF1 Protects Against Acute Liver Toxicity After Doxorubicin Treatment via the AMPK/NRF2 Pathway. Front Pharm. 13. 10.3389/fphar.2022.940406. PMC9468578 36110535

[R88] Ye H, Wu L, Liu Y, 2024. Iron metabolism in doxorubicin-induced cardiotoxicity: From mechanisms to therapies. Int. J. Biochem. Cell Biol 174, 106632. 10.1016/j.biocel.2024.106632. 39053765

[R89] Yeerkenbieke B, Li Y, Kiessling F, , 2025. Understanding the action mechanisms and safety of nanoparticles with functional toxicogenomics. Nanomedicine 20, 2405–2409. 10.1080/17435889.2025.2523733. 40567138 PMC12490377

[R90] Yimit A, Adebali O, Sancar A, , 2019. Differential damage and repair of DNA-adducts induced by anti-cancer drug cisplatin across mouse organs. Nat. Commun 10, 309. 10.1038/s41467-019-08290-2. 30659176 PMC6338751

[R91] Zobeydi AM, Mousavi Namavar SN, Sadeghi Shahdani M, , 2025. Mitigating doxorubicin-induced hepatotoxicity in male rats: the role of aerobic interval training and curcumin supplementation in reducing oxidative stress, endoplasmic reticulum stress and apoptosis. Sci. Rep 15, 6604. 10.1038/s41598-025-91133-6. 39994295 PMC11850886

